# Supplementary material for: ’We weren't checked in on, nobody spoke to us’: an exploratory qualitative analysis of two focus groups on the concerns of ethnic minority NHS staff during COVID-19
Source: BMJ Open. 2021 Dec 31;11(12):e053396. doi: 10.1136/bmjopen-2021-053396 (PMC8720640; doi:10.1136/bmjopen-2021-053396)
Supplement: Supplementary data [file bmjopen-2021-053396supp001.pdf]

Focus group question guide

General

1. In general terms, how do you think being from BAME backgrounds has impacted upon your experiences during COVID?

Inside work experiences

2. Thinking of your experiences in your workplace, during COVID have you felt that you were treated differently from non-BAME staff? Is that by management and / or fellow workers? Why do you think that? Can you provide examples?

3. How do you think that your background has influenced your interactions with other staff members? What form has that taken? Why do you think that?

4. Did the way you were treated by your colleagues change during COVID compared to "normal times" (i.e., pre-COVID times)? If yes, how did it change? Can you give examples?

5. Did you notice any differences in the experiences of BAME and non-BAME staff during COVID? If yes, what form did they take?

6. Do you feel that you were given tasks/responsibilities that put you more at risk of contracting COVID than your non-BAME colleagues? If so, how did you respond in these situations? What was the subsequent response of the line manager requesting these tasks?

7. Do you feel others are responding to an aspect of your background in your dealings with them? If yes, what aspect do you think that is? (e.g., your ethnic group, physical appearance, culture, faith, etc).

8. Do you feel that your BAME background has affected the way you are treated by patients during COVID? If yes, please explain.

9. How well did you feel supported by your colleagues generally during COVID? If supported, how? If not supported, what support was absent and why do you think that was the case?

Outside work experiences

10. How has your home life changed during COVID? If yes, how exactly?

11. Has this affected your work life? If yes, how exactly?

12. Has there been an impact upon your children and other family members arising from their BAME background? If yes, please explain.

13. Have you experienced any changes in public life (e.g., on public transport) that you think is linked to your BAME background? If yes, please explain.

14. Has your work life affected your home life in any way? Please explain how.

15. If asking other BAME colleagues about their COVID experiences, what are the important questions to ask them?

16. Is there anything we didn't talk about which you think it would be useful to know?
