# Supplementary material for: ’We weren't checked in on, nobody spoke to us’: an exploratory qualitative analysis of two focus groups on the concerns of ethnic minority NHS staff during COVID-19
Source: BMJ Open. 2021 Dec 31;11(12):e053396. doi: 10.1136/bmjopen-2021-053396 (PMC8720640; doi:10.1136/bmjopen-2021-053396)
Supplement: Supplementary data [file bmjopen-2021-053396supp002.pdf]

MeCareNWL survey module on the experiences of healthcare staff from minority ethnic backgrounds

Q1 Do you feel your employer acknowledged BAME staff's increased vulnerability to COVID-19?

- ◯ Yes
- ◯ No
- ◯ Don't know

Q1a To what extent do you feel your employer has acknowledged BAME staff's increased vulnerability to COVID-19?

No acknowledgement — Fully acknowledged vulnerability

	0	10	20	30	40	50	60	70	80	90	100
1.											

Q2 Do you feel your employer has accommodated BAME staff's increased risk from COVID-19?

- ◯ Yes
- ◯ No
- ◯ Don't know

Q2b What do you feel your employer **could have done** to accommodate BAME staff's increased risk from COVID-19?

○ 1. ____

○ 2. ____

○ 3. ____

○ 4. ____

○ 5. ____

○ 6. ____

○ 7. ____

Q3 Did your employer take actions that made you feel safer in terms of your vulnerability to the virus?

○ Yes

○ No

○ Not sure

Q3a What actions did your employer take that made you feel safer in terms of your vulnerability to the virus? (please select all that apply)

☐ Allowed me to work from home

☐ Provided me with adequate PPE

☐ Moved me to work in lower-risk wards / away from the front line

☐ Other (please state)

____

Q4 Do you feel able to speak to your manager about concerns regarding your heightened vulnerability to COVID-19 due to your ethnicity?

- ◯ Yes
- ◯ No
- ◯ Don't know

Q4a To what extent do you feel able to speak to your manager about concerns regarding your vulnerability to COVID-19?

	Unable to voice my concerns	Comfortable and able to speak freely
	0 10 20 30 40 50 60 70 80 90 100	
1.		

Q5 Did you report any concerns related to your health vulnerability associated with your ethnicity to your managers?

- ◯ Yes
- ◯ No
- ◯ Don't know

Q5a To what extent did you feel that your manager understood your concerns related to your health vulnerability associated with your ethnicity?

	No understanding at all	Full understanding
	0 10 20 30 40 50 60 70 80 90 100	
1.		

Q6 If you worked from home during COVID-19, do you feel that your BAME status affected how much your manager trusted you while you were working from home?

- ◯ Yes
- ◯ No
- ◯ Don't know
- ◯ Didn't work from home

Q6a To what extent do you feel that your manager's trust of you while working from home is due to your ethnicity?

Less trusting Because I'm BAME — More trusting because I'm BAME

0 10 20 30 40 50 60 70 80 90 100

1.

Q7 Do you feel that you have had to take risks greater than you are comfortable with?

- ◯ Yes
- ◯ No
- ◯ Don't know

Q7a How frequently did you feel you had to take risks greater than you were comfortable with in your workplace?

Never — Very frequently

0 10 20 30 40 50 60 70 80 90 100

1.

Q8 Did BAME staff have to work in high-risk areas in your Trust / local authority / department / practice?

- Yes
- No
- Don't know

Q8a How common do you feel that the experience of BAME staff working in high-risk areas was in your Trust / local authority / department / practice?

Very rare — Very common

0 10 20 30 40 50 60 70 80 90 100

1.

Q9 Do you feel that your ethnic background played a role in your ability to obtain PPE?

- Yes
- No
- Don't know

Q9a To what extent do you feel that your ethnic background has played a role in your ability to obtain PPE?

Made it harder to obtain PPE — Made it easier to obtain PPE

0 10 20 30 40 50 60 70 80 90 100

1.

Q10 Within your workplace, which of the following provided you with a source of support when needed? (please select all that apply)

- ▢ Manager
- ▢ BAME colleagues
- ▢ Non-BAME colleagues
- ▢ Trust staff support service
- ▢ No source of support
- ▢ Other (please specify) ____

Q11 Did you feel that patients' level of appreciation for you and other BAME staff was different from their level of appreciation for non-BAME staff?

- ◯ Yes
- ◯ No
- ◯ Don't know

Q11a How do you feel that patient's level of appreciation for staff differed between BAME staff and non-BAME staff?

	Less appreciation for BAME staff than non-BAME staff										More appreciation for BAME staff than non-BAME staff
	0	10	20	30	40	50	60	70	80	90	100
1.											

Q12 Did you **expect** to experience increased prejudice associated with your ethnic background as a result of COVID-19 **in your workplace**?

- ◯ Yes
- ◯ No
- ◯ Don't know

Q13 Did you **actually** experience increased prejudice associated with your ethnic background as a result of COVID-19 **in your workplace**?

- ◯ Yes
- ◯ No
- ◯ Don't know

Q14 Did you **expect** to experience increased prejudice associated with your ethnic background as a result of COVID-19 **in the community**?

- ◯ Yes
- ◯ No
- ◯ Don't know

Q15 Did you **actually** experience increased prejudice associated with your ethnic background as a result of COVID-19 **in the community**?

- ◯ Yes
- ◯ No
- ◯ Don't know

Q16 What practical suggestions do you have that would help you feel **heard** by management?

____

Q17 What practical suggestions do you have that would help you feel **safer** in your workplace?

____
