# Supplementary material for: ’We weren't checked in on, nobody spoke to us’: an exploratory qualitative analysis of two focus groups on the concerns of ethnic minority NHS staff during COVID-19
Source: BMJ Open. 2021 Dec 31;11(12):e053396. doi: 10.1136/bmjopen-2021-053396 (PMC8720640; doi:10.1136/bmjopen-2021-053396)
Supplement: Author's manuscript [file bmjopen-2021-053396.draft_revisions.pdf]

BMJ Open is committed to open peer review. As part of this commitment we make the peer review history of every article we publish publicly available.

When an article is published we post the peer reviewers' comments and the authors' responses online. We also post the versions of the paper that were used during peer review. These are the versions that the peer review comments apply to.

The versions of the paper that follow are the versions that were submitted during the peer review process. They are not the versions of record or the final published versions. They should not be cited or distributed as the published version of this manuscript.

BMJ Open is an open access journal and the full, final, typeset and author-corrected version of record of the manuscript is available on our site with no access controls, subscription charges or pay-per-view fees (http://bmjopen.bmj.com).

If you have any questions on BMJ Open's open peer review process please email info.bmjopen@bmj.com

BMJ Open

"We weren't checked in on, nobody spoke to us":Understanding ethnic minority NHS staff concerns during COVID-19 to develop a dedicated survey module

Journal:	*BMJ Open*
Manuscript ID	bmjopen-2021-053396
Article Type:	Original research
Date Submitted by the Author:	11-May-2021
Complete List of Authors:	Jesuthasan, Jehanita; Imperial College London Faculty of Medicine, Department of Brain Sciences<br>Powell, Richard A; Imperial College London School of Public Health, Department of Primary Care and Public Health; NIHR Collaboration for Leadership in Applied Health Research and Care for Northwest London<br>Burmester, Victoria; Imperial College London Faculty of Medicine, Department of Brain Sciences<br>Nicholls, Dasha; Imperial College London Faculty of Medicine, Department of Brain Sciences; NIHR Collaboration for Leadership in Applied Health Research and Care for Northwest London
Keywords:	COVID-19, MENTAL HEALTH, Health & safety < HEALTH SERVICES ADMINISTRATION & MANAGEMENT, OCCUPATIONAL & INDUSTRIAL MEDICINE

SCHOLARONE™
Manuscripts

I, the Submitting Author has the right to grant and does grant on behalf of all authors of the Work (as defined in the below author licence), an exclusive licence and/or a non-exclusive licence for contributions from authors who are: i) UK Crown employees; ii) where BMJ has agreed a CC-BY licence shall apply, and/or iii) in accordance with the terms applicable for US Federal Government officers or employees acting as part of their official duties; on a worldwide, perpetual, irrevocable, royalty-free basis to BMJ Publishing Group Ltd ("BMJ") its licensees and where the relevant Journal is co-owned by BMJ to the co-owners of the Journal, to publish the Work in this journal and any other BMJ products and to exploit all rights, as set out in our licence.

The Submitting Author accepts and understands that any supply made under these terms is made by BMJ to the Submitting Author unless you are acting as an employee on behalf of your employer or a postgraduate student of an affiliated institution which is paying any applicable article publishing charge ("APC") for Open Access articles. Where the Submitting Author wishes to make the Work available on an Open Access basis (and intends to pay the relevant APC), the terms of reuse of such Open Access shall be governed by a Creative Commons licence – details of these licences and which Creative Commons licence will apply to this Work are set out in our licence referred to above.

Other than as permitted in any relevant BMJ Author's Self Archiving Policies, I confirm this Work has not been accepted for publication elsewhere, is not being considered for publication elsewhere and does not duplicate material already published. I confirm all authors consent to publication of this Work and authorise the granting of this licence.

For peer review only

"We weren't checked in on, nobody spoke to us": Understanding ethnic minority NHS staff concerns during COVID-19 to develop a dedicated survey module

Jehanita Jesuthasan[1]

Richard A Powell[2, 3]

Victoria ML Burmester[1]

Dasha Nicholls[1, 3]

[1] Imperial College London Faculty of Medicine, Department of Brain Sciences, London, UK

[2] Imperial College London School of Public Health, Department of Primary Care and Public Health, London, UK

[3] National Institute for Health Research Collaboration for Leadership in Applied Health Research and Care for Northwest London, London, UK

Correspondence to:

Dr Dasha Nicholls

Mailing address: Department of Brain Sciences, Faculty of Medicine, Imperial College London, Burlington Danes, The Hammersmith Hospital, Du Cane Road, London W12 0NN

Email: d.nicholls@imperial.ac.uk

Tel: +44 (0)20 3313 8436

Word count: 3816

For peer review only - http://bmjopen.bmj.com/site/about/guidelines.xhtml

For peer review only

Statement of ethics approval: The MeCare study received ethical approval from the Health Research Authority (IRAS Project ID 290383), which included permission to co-create a module addressing questions specifically for ethnic minority populations with input from Patient and Public Involvement (PPI) colleagues, project partners and other experts by experience. Participants gave their informed consent before taking part.

Funding: This report is independent research funded by the National Institute for Health Research North West London Applied Research Collaboration (ARC) and by the Imperial College COVID-19 Research Fund grant number P88408. The views expressed in this publication are those of the authors and not necessarily those of the National Institute for Health Research or the Department of Health and Social Care.

Competing interests: None declared.

Data sharing statement: No additional data are available.

Author contributions: JJ, RAP, VB, and DN conceptualised the study. RAP conducted the focus groups. JJ, RAP, and VB analysed the data. JJ wrote the draft with support from RAP, VB, and DN. RAP, VB, and DN managed the overall design of the study.

Non-author contributors: John Norton and Sandra Jayacodi, partners of the National Institute for Health Research ARC Northwest London PPI Initiative, verified data interpretation and reviewed the final manuscript.

Acknowledgements: We would like to thank the participants for taking part in this research and sharing their experiences.

Figures

Figure 1: Thematic Experiences of Ethnic Minority Healthcare Staff During COVID-19

Supplementary Materials

For peer review only - http://bmjopen.bmj.com/site/about/guidelines.xhtml

1. Interview guide for the focus groups
2. Survey module created based on the focus groups

For peer review only

For peer review only - http://bmjopen.bmj.com/site/about/guidelines.xhtml

ABSTRACT

Objective

To examine the multi-faceted, lived-experience impact of COVID-19 on ethnic minority healthcare staff to co-create a module of questions for follow-up online surveys on the wellbeing of healthcare staff during the pandemic.

Design

A cross-sectional design using two online focus groups among ethnic minority healthcare workers who worked in care or supportive roles in a hospital, community health or GP surgery setting for at least 12 months.

Participants

Thirteen healthcare workers (11 female) aged 26 to 62 years from diverse ethnic minority backgrounds, ten working in clinical roles.

Results

Five primary thematic domains emerged: 1) *viral vulnerability*, centring around perceived individual risk and vulnerability perceptions; 2) *risk assessment*, comprising of pressures to comply, perception of a tick-box exercise, and issues with risk and resource stratification; 3) *interpersonal relations in the workplace*, highlighting deficient consultation of ethnic minority staff, cultural insensitivity, need for support, and collegiate judgement; 4) *lived experience of racial inequality*, consisting of job insecurity and the exacerbation of systemic racism and its emotional burden; 5) *community attitudes*, including public prejudice and judgement, and patient appreciation.

For peer review only

Conclusions

Our novel study has shown ethnic minority NHS staff have experienced COVID-19 in a complex, multi-dimensional manner. Future research should enumerate the extent to which these varied thematic experiences are shared among ethnic minority NHS workers so that more empathetic and supportive management and related occupational practices can be instituted.

For peer review only

ARTICLE SUMMARY

Strengths and limitations of this study

- This is one of the first studies to examine qualitatively the experiences of ethnic minority healthcare staff during the COVID-19 pandemic.
- It provides a basis for the creation of a survey module to examine the experiences of ethnic minority healthcare staff across north-west London.
- Due to the self-selected nature of the sample, the experiences of participants may not be representative of the ethnic minority healthcare workforce as a whole.
- Given its exploratory nature, the sample size was potentially insufficient to achieve data saturation.

KEYWORDS: COVID-19; Mental Health; Occupational Health; Health and Safety

For peer review only - http://bmjopen.bmj.com/site/about/guidelines.xhtml

INTRODUCTION

COVID-19 has adversely impacted the occupational roles and physical and mental wellbeing of healthcare staff[1]. Many have experienced disrupted support structures, redeployment to areas outside their professional training[2]—sometimes engaging in tasks that transgress individual moral consciences and values[3]—limited resources and vital medical equipment[4], and unprecedented patient service demand amid rapidly changing care guidelines[5]. Staff are not only vulnerable to contracting the virus but at risk of work stress, moral injury, and mental ill-health, including clinical depression, post-traumatic stress disorder, substance misuse, and suicide[6].

Ethnic minority care staff have been disproportionately affected by the pandemic's clinical impact[7].The impact is multi-faceted: economic, social, attitudinal and cultural, as well as occupational, physical and psychological[8 9]. Understanding these broader impacts can facilitate a more holistic and empathetic understanding of the lived experiences of COVID-19 among health care staff from ethnic minorities, generating greater awareness of health-seeking behaviour in and outside the workplace, and perceived impediments to seeking and receiving support[10 11]. Increased understanding can also inform public health campaigns addressing, for example, disinclination to accept vaccines[12-14].

The MeCare study in Northwest (NW) London, collaborating with partners in the larger *NHS Check* study[15 16], is longitudinally examining the mental health and wellbeing of National Health Service (NHS) staff (including general practitioners) in NW London—where 49% of NHS staff are from ethnic minority backgrounds[17]—and exploring how to support staff during pandemics. This paper reports on the co-creation of a module of questions targeted towards staff from ethnic minority backgrounds as part of follow-up online surveys.

For peer review only - http://bmjopen.bmj.com/site/about/guidelines.xhtml

METHOD

Participants

We explored the experiences of ethnic minority healthcare workers selected through purposive sampling. Participants were recruited through Patient and Public Involvement (PPI) networks and promotional materials circulated among NHS trusts. Participants were adults from an ONS[18] ethnic minority background who worked in care or supportive roles in a hospital, community health or GP surgery setting for at least 12 months.

Data collection

Two 2-hour online focus groups—shown to generate idea diversity comparable to in-person equivalents[19]—were held using Microsoft Teams (MT) and facilitated by RAP, with participants offered the choice of having their session cameras live or not. Both groups followed the study interview guide (Supplementary File 1), which was reactively adapted, in terms of questioning order and prompts, and field notes were taken on key points.

Data analysis

Discussions were transcribed verbatim using MTs' automatic caption generation software and manually checked for errors. RAP, VB and JJ undertook independent thematic analysis using the template analysis approach[20], combining deductive and inductive analyses. The study team subsequently met to reach consensus on emergent themes and sub-themes. To ensure the veracity of data interpretation, the findings and draft survey questions were validated by member checking with a sample of group participants[21].

Patient and public involvement

Participants were a sample of the MeCare study's target population and recruited through PPI representatives. JN and SJ are public partners of the Applied Research Collaboration in NW London and provided feedback on data interpretation.

For peer review only - http://bmjopen.bmj.com/site/about/guidelines.xhtml

RESULTS

The final sample included 13 healthcare workers (11 female) with a mean age of 42.7 years (SD = 10.3 years). Ethnically the sample was comprised of: Eastern European (n = 1), Indian (n = 2), Mixed White and Black Caribbean (n = 1), Caribbean (n = 1), Mixed White and Asian (n = 1), African (n = 2), Arab (n = 1), Bangladeshi (n = 1), Asian Other (n = 2), and Other, in this instance Mixed North African and Eastern European, (n = 1). Ten worked in clinical roles; three administrative.

The five primary thematic domains that emerged were: viral vulnerability, risk assessment, interpersonal relationships in the workplace, lived experience of racial inequality, and community attitudes (Figure 1).

Figure 1: Themes and subthemes identified through thematic analysis

1) Viral vulnerability

a) Perceived individual risk

Participants were sensitive to their increased risk of infection compared to non-ethnic minority colleagues in the same roles. They discussed the impact of this heightened risk on their emotional wellbeing, reporting personal anxiety and emotional distress.

> P12: "I think most upsetting was the initial kind of response and risk stratifications and the deaths of people I knew or knew by indirect means."

Participants also expressed frustration that individual vulnerability was insufficiently acknowledged within their teams and organisations, a shortcoming one participant suggested

For peer review only - http://bmjopen.bmj.com/site/about/guidelines.xhtml

was associated with the fact that it was ethnic minorities rather than the non-minority population who were affected.

> P2: "If the prevalence and incidence was affecting the White majority population more than the Black population, maybe something could be done differently."

b) Vulnerability misperceptions

Several participants expressed frustration about fundamental misunderstandings regarding the reasons for increased infection risk among ethnic minorities: in public discourse and government reports[22], differences in viral vulnerability were frequently attributed to individual factors (personal hygiene practices, individual obesity). Participants felt insufficient attention was paid to structural determinants, from exposure differentials to unconscious racism.

> P3: "That research was basically saying that [ethnic minorities] are at more risk because they are obese, they have got Type 2 diabetes, they're living in overcrowded situations and all that. […] They did not consider that this could be just because we are exposed more."

> P13: "It's just being aware that it's not entirely biological, the risk that they face. Some of the reasons why we saw people from BAME [Black, Asian

For peer review only - http://bmjopen.bmj.com/site/about/guidelines.xhtml

and minority ethnic] background suffer a lot more was because of their social circumstances as well.”

2) Risk assessment

a) Tick-box exercise

Many participants felt risk assessment exercises had not been treated seriously by their managers and organisations. Although some felt their employer accommodated their identified increased risk, many reported not seeing tangible protective actions for vulnerable ethnic minority staff. By not emphasising the importance of completing risk assessments, and taking minimal action following them, management made staff feel the assessment was a tick-box exercise rather than a considered strategy to reduce staff risk.

> P3: “I wasn’t able to understand what the point of that risk assessment was and if somebody was found to be high risk population, whether something different was done for that person.”

b) Pressure to comply

Despite awareness of ethnic minority communities’ COVID-19 vulnerability, participants reported feeling under pressure to continue frontline working. This arose both from managers and an innate sense of duty, with a sense of guilt associated with not seeing in-person patients.

For peer review only - http://bmjopen.bmj.com/site/about/guidelines.xhtml

> P11: "I did feel quite guilty about not being at work seeing patients. […] Every Thursday for a while [my neighbours] were all standing outside clapping and I didn't go outside. I stayed inside and hid because I felt like a complete fraud."

Participants frequently felt they had minimal choice but to continue working in high-risk positions.

> P5: "You kind of feel you're obligated to follow through your duties, but there still is that major risk of putting yourself or your health at risk."

In terms of extrinsic pressure, the demands on NHS frontline staff due to surges in COVID-19 cases[23], combined with staffing shortages[24] and the high proportion of ethnic minority NHS staff[17], limited the alternatives to positioning ethnic minority staff in frontline care provision. Consequently, many reported no choice but to maintain their position on high-risk wards—even when Personal Protective Equipment (PPE) was in short supply—and being pressured to return to work shortly after COVID-19 diagnoses.

> P5: "If you pull out all the people on the frontline, then who's going to actually do the work? Who's gonna fill in those gaps?"

> P12: "They were desperate to get people on the ground and there was this whole thing about 'it's a respiratory virus and it only lasts 2 weeks max'. So,

For peer review only - http://bmjopen.bmj.com/site/about/guidelines.xhtml

many members of staff were harassed, told ‘you have to go back, your two weeks are up’.”

P4: “Your personal risk doesn’t matter, the managers are managers, they’re there to get the job done, to apply the pressure coming from above. So, if you’re a nurse in a unit that is short-staffed and you turn up to work and they don't have [the necessary PPE], you either get on with your job knowing that you're at high risk, or you become a problem.”

c) Stratified risk and resources

Several participants identified flaws in the risk and resultant resources stratification, feeling the organisation’s workplace hierarchy was imposed on the risk assessment. They contended the distribution of PPE was based on professional hierarchy rather than individual vulnerability.

P4: “The distribution of PPE seems to have followed a hierarchical structure. So, medics are walking around with 3M rubber fit-sealed masks, although they don't spend that much time in COVID areas, while some of the Filipino nurses are struggling to find a 3M mask that fits them. And then when it comes to risk assessing the domestic staff, they’re an afterthought.”

For peer review only - http://bmjopen.bmj.com/site/about/guidelines.xhtml

3) Interpersonal relations in the workplace

a) Deficient consultation

The perception of blame for increased vulnerability to COVID-19 being ascribed to ethnic minority communities and a disregard for the role of structural factors fuelled belief in a lack of understanding of ethnic minority experiences. Moreover, the view that attempts to remedy this deficiency were lacking reinforced the sense that larger racial issues were disregarded and thereby unsolved. Participants reported ethnic minorities were excluded from conversations concerning them—such as the construction of risk assessments—and felt the impacts of their vulnerability on their wellbeing were disregarded within organisations.

> P1: "We weren't checked in on, nobody spoke to us to see if we were okay, how we felt about it. It just wasn't really made a big deal of. It wasn't something that mattered."

> P2: "The issue of not being heard, as somebody from the BAME community. 'Cause I felt like, up to now, nobody's really asked me 'What do you want? What can we do to make you feel protected?' So, I'm aware that the risk assessment was imposed by other people from other organisations. […] It was again that issue of them discussing about us without us."

For peer review only - http://bmjopen.bmj.com/site/about/guidelines.xhtml

b) Cultural insensitivity

The disconnect between ethnic and non-ethnic minority staff was associated with difficulties sharing concerns with non-minority supervisors. Several participants cited cultural differences and the associated lack of understanding of ethnic minority staff's experiences, needs, and problems as the principal barrier to discussing concerns with non-ethnic minority managers. Indeed, it was suggested that a common background with one's supervisor would facilitate communication and increase feelings of being supported.

> P4: "I was staying in the trust hotel and went out for a walk […] I was subsequently stopped and searched by police, assaulted. Trying to explain that to my manager resulted in the eye-rolling from them, and them not really understanding the consequences of that."

> P3: "It is always much easier to explain to people who are from your own cultural background or somebody who has experienced the same kind of thing rather than actually going to your manager who has got no idea what you're talking about."

For example, the difficulty obtaining hair caps, needed by ethnic minority staff who do not wash their hair daily, highlighted the impact of under-representation of such staff in senior positions and ethnic minority staff's ability to perform their jobs.

For peer review only - http://bmjopen.bmj.com/site/about/guidelines.xhtml

c) Need for support

Several participants stated they did not feel adequately supported by their employer and their manager during the pandemic. Some felt particularly unsupported when their supervisor was from a non-ethnic minority background, making it difficult to know their concerns related to ethnicity were heard. For participants who felt they had organisational support, it was seen as improving their wellbeing and may have mitigated the effects of racial injustices they were increasingly aware of and subjected to during the pandemic.

> P3: "Will [ethnic minority staff] feel more comfortable and more supported if they have somebody appointed that they can approach if they have any issues? […] Will they feel more comfortable if that person is from the same cultural background?"

> P11: "I think people were quite protective of me [because I'm ethnic minority] and I personally didn't find that paternalistic or patronising. I felt cared for."

d) Collegiate judgement

Several participants were wary of special treatment being accorded to them, fearing it might negatively impact how they are viewed by non-ethnic minority colleagues. It was suggested some ethnic minority staff may have been reluctant to be risk-stratified and made to work from home for fear of being negatively judged by team members.

For peer review only - http://bmjopen.bmj.com/site/about/guidelines.xhtml

> P11: “There were a significant minority of people who, although my recommendation was that they should work from home, they chose to not follow guidance and they wanted to see patients face to face. […] I suspect that might have been because they were worried about their position in the team and about bullying.”

Participants also noted that, in some cases, the special attention given to ethnic minority individuals was experienced as discrimination and a cause for bullying.

> P11: “There were a lot of people who were BAME who did feel that there was a spotlight being shone upon them. […] A lot of them didn't feel that that was positive. They felt that it could be seen as quite discriminatory, as them being given special privileges.”

Moreover, several participants were defensive of the reasons for doing their jobs, emphasising their interest in, and passion for, their work and denying ulterior motives, such as hopes to receive special treatment.

> P9: “It’s work that the BAME group tends to have interest in, and we do it with a love for it, rather than wanting to be specially treated. I think sometimes when there's a lot of talk about BAME groups, it can be a little bit patronising because you feel that's a career that you've always wanted to do.”

For peer review only - http://bmjopen.bmj.com/site/about/guidelines.xhtml

This further highlights the fear of one's job performance being judged by colleagues based on ethnicity.

4) Lived experience of racial inequality

a) Exacerbation of systemic racism and its emotional burden

There was a strong sense that existing structural workplace inequality and the emotional burden of racial injustice were exacerbated during the pandemic. Participants felt ethnic minority staff have a distinct disadvantage in career advancement, noting they had to work harder than non-minority counterparts to progress their careers. Crucially, several participants felt that increased COVID-19 vulnerability compounded career advancement challenges, and that the pandemic provided an opportunity for unconscious bias among management to manifest in career advancement opportunities given to non-ethnic minority staff.

> P11: "I think that there's something similar [to being female] about being BAME compared to being White, where you know you have to work so much harder to get where you want to go to. […] Then there's COVID affecting us in a more serious way, […] it's another disadvantage."

> P4: "There are at least a few cases of people in the same staff group occupying two bandings. And unfortunately, BAME staff at one, slightly lower, band. And

For peer review only - http://bmjopen.bmj.com/site/about/guidelines.xhtml

> management have taken it upon themselves to use the chaos that's going on to promote the same types of people that always get promoted."

Additionally, participants noted the emotional burden of the pandemic is especially high among ethnic minority staff and that the Black Lives Matter movement[25] compounded feelings of racial injustice evoked in the workplace during the pandemic and highlighted structural, societal problems.

> P1: "All of the Black Lives Matter stuff that was going on, and George Floyd and the protests, the whole attitude around them in the workplace was just exacerbating people's feelings and made it very difficult. It felt like you were being attacked from all sides."

b) Job insecurity

Participants also reported the perception that working from home may threaten their job due to racial inequality in the workplace and that managers were less trusting of ethnic minority staff working from home than non-ethnic minority counterparts.

> P11: "I found that the drawback [of working from home] was I wasn't getting my face seen [at work]. […] I actually almost felt that my job was threatened, I became very anxious and very insecure about my place within the team."

For peer review only - http://bmjopen.bmj.com/site/about/guidelines.xhtml

> P9: “[At the time I was working from home] I had a colleague I was working with who felt that that pressure [from managers] was given to the ethnic minority group because we were not trusted to be doing what we should be doing.”

5) Community attitudes

a) Public prejudice and judgement

The racial injustice experienced by ethnic minority staff in their workplace also occurred in the community, where participants reported witnessing racist slurs against East Asians and anticipating an increase in such attitudes towards themselves.

> P10: “I've felt a lot more worried about having sort of racial attacks or racial comments pointed in my direction, given some of the narrative around COVID, such as the ‘Chinese Virus’.”

Additionally, the ethnicity-based judgements participants reported receiving from the public reinforced their understanding that ethnic minority staff were seen as less than non-ethnic minority colleagues in society.

> P5: “You feel like although you're coming to do something positive but at the same time other people might be making all sorts of judgments as to what you're doing.”

For peer review only - http://bmjopen.bmj.com/site/about/guidelines.xhtml

P5: "One of my neighbours said I shouldn't be going outside, so, I said 'No, I work for the NHS' and automatically his response was 'Oh! Are you a cleaner?'."

b) Patient appreciation

Despite racial prejudices experienced in the community, participants noted patients showed an increased recognition of the value ethnic minority communities bring to the NHS and felt this increased patient appreciation was reflective of a broader attitudinal shift towards their key role.

P6: "[Patients] were very happy to be seen, happy to be taken care of, they were politer. They really appreciated what we were doing."

P1: "I think there was a shift in [patients'] attitude towards NHS staff in general, which was refreshing."

In contrast, others were sceptical of whether this recognition was indicative of a real attitudinal shift, recalling past NHS criticism[26]. Moreover, participants highlighted a disconnect between the public's appreciation of the NHS, expressed in weekly street clapping, and practical acknowledgement of the risk to which NHS staff are regularly exposed.

For peer review only - http://bmjopen.bmj.com/site/about/guidelines.xhtml

P13: “When people would clap for the NHS I would think ‘Oh my God they’re clapping. What are they talking about? Do they even know what happens inside?’.”

DISCUSSION

This exploratory group process examined the lived experiences of healthcare staff from ethnic minority backgrounds during COVID-19 to develop an ethnically empathetic module for follow-up survey stages in the MeCare study (Supplementary File 2). The five primary themes that emerged from our research have highlighted key areas of concern and neglect that need to be enumerated and investigated further to appreciate the extent to which they are shared by ethnic minority staff.

These concerns are consistent with research regarding the depression, anxiety, and stress associated with perceived COVID-19 risk and the pandemic’s impact on ethnic minority communities[27-29]. The perceived pressure to work despite individual risk can significantly impact staff’s work performance and mental health. For example, presenteeism—individuals presenting at work but operating sub-optimally due to health issues—can have serious consequences if it leads to poor, slow, or incorrect decision-making[30]. Moreover, Shah et al.[31] reported that during the pandemic, ethnic minority staff faced dilemmas around fulfilling their duty and continuing to deliver patient clinical care or exercising mitigating actions to avoid high-risk environments.

Ethnic minority staff’s mental health is likely to also be impacted by a disconnect between ethnic minority and non-minority staff. Indeed, post-trauma social support from managers influences staff’s long-term mental health status[32 33], and discussion of ethnic minority staff’s experiences during COVID-19 with non-minority supervisors can help foster

For peer review only - http://bmjopen.bmj.com/site/about/guidelines.xhtml

improved attitudes towards mental health in the workplace[34]. The disconnect was manifested in fear of bullying, which is consistent with evidence that ethnic minority staff are more likely than non-minority colleagues to experience staff bullying[35 36]. Additionally, participants' sense that the pandemic exacerbated existing emotional burdens regarding systemic racism is consistent with an interplay between racism and the pandemic[37]. This may also contribute to longer-term mental ill-health—including the psychological consequences of trauma exposure[32 38 39]—among ethnic minority staff[40]. On a positive note, however, the public's acknowledgement of work undertaken by staff during the pandemic helps promote staff's resilience, thereby protecting their mental health[41].

Participants' accounts also suggest a perception that nurses and domestic staff—who are more likely to be from ethnic minority backgrounds[42] and are particularly vulnerable to infection due to their physical proximity to patients when providing care[43]—struggled to secure adequate PPE, which was more easily available for senior staff. The perception that PPE distribution was based on professional hierarchy is important. Previous research has found that, among healthcare workers, the incidence of anxiety was highest among non-medical healthcare staff, possibly because they had less first-hand information about the disease and less training on infection control measures and PPE use[44]. Additionally, adequate consultation of ethnic minority staff is especially important given they are already less likely than non-minority counterparts to voice their opinions and raise concerns about inadequate PPE, increasing the likelihood of their needs being overlooked[36 45].

Racial inequalities were also reported in career advancement and treatment by managers. This is consistent with reports that ethnic minority staff have been restricted to certain NHS roles due to inequalities in career development and workplace and societal discrimination, rendering them more likely to work in critical specialties and services during the pandemic[46]. Moreover, ethnic minority staff are also more likely than non-ethnic minority

For peer review only - http://bmjopen.bmj.com/site/about/guidelines.xhtml

staff to experience excessive scrutiny and punishment[47] and discrimination[48] from their managers.

In terms of study limitations, the sample was self-selected and, as such, participants were likely to feel more strongly about their experience of the pandemic compared to the ethnic minority healthcare workforce as a whole. However, participants were purposively sought for their opinions to gain broad insights into this neglected area to inform further inquiry, rather than generating representative data. Second, there is the possibility that the facilitator's race (non-minority ethnic) biased the data by inhibiting open discussion[49]. However, the facilitator has over 20 years' experience of health research in diverse settings including low- and middle-income countries, and JJ, who was present in both focus groups, is minority ethnic. In addition, discussions were frank and unimpeded, and evidence indicates the impacts of the interviewer's ethnicity are minimal[50]. Third, the sample size was potentially insufficient to achieve thematic saturation as this was an exploratory exercise to highlight largely unexamined thematic areas that are currently being further researched. To minimize the possibility of the authors' invalid interpretation of the data, the study used respondent validation among a sample of group participants.

Our novel study has clearly shown that ethnic minority NHS staff have experienced COVID-19 in a complex, multi-dimensional manner. The physical and psychological impact of the virus has been overlayed by wider occupational, attitudinal (including anticipatory racism) and socio-cultural experiences. A need exists to understand the extent to which these varied experiences are shared among ethnic minority NHS workers so more empathetic and supportive management and related occupational practices can be instituted.

For peer review only - http://bmjopen.bmj.com/site/about/guidelines.xhtml

References

1. Gilleen J, Santaolalla A, Valdearenas L, et al. The impact of the COVID-19 pandemic on the mental health and wellbeing of UK healthcare workers. *MedRxiv* [Preprint]. 2020. (accessed March 2021).
2. Vera San Juan N, Camilleri M, Jeans JP, et al. Redeployment and training of healthcare professionals to Intensive Care during COVID-19: a systemic review. *MedRxiv* [Preprint]. 2021. (accessed March 2021).
3. Newdick C, Sheehan M, Dunn M. Tragic choices in intensive care during the COVID-19 pandemic: on fairness, consistency and community. *J Med Ethics* 2020;46(10):646-51. doi: 10.1136/medethics-2020-106487
4. Supady A, Curtis JR, Abrams D, et al. Allocating scarce intensive care resources during the COVID-19 pandemic: practical challenges to theoretical frameworks. *The Lancet Respiratory Medicine* 2021;9(4):430-34. doi: 10.1016/s2213-2600(20)30580-4
5. Vindrola-Padros C, Andrews L, Dowrick A, et al. Perceptions and experiences of healthcare workers during the COVID-19 pandemic in the UK. *BMJ Open* 2020;10(11):e040503. doi: 10.1136/bmjopen-2020-040503
6. Greenberg N. Mental health of health-care workers in the COVID-19 era. *Nat Rev Nephrol* 2020;16(8):425-26. doi: 10.1038/s41581-020-0314-5
7. Shields A, Faustini SE, Perez-Toledo M, et al. SARS-CoV-2 seroprevalence and asymptomatic viral carriage in healthcare workers: a cross-sectional study. *Thorax* 2020;75(12):1089-94. doi: 10.1136/thoraxjnl-2020-215414
8. Jaspal R, Lopes B. Discrimination and mental health outcomes in British Black and South Asian people during the COVID-19 outbreak in the UK. *Mental Health, Religion & Culture* 2021;24(1):80-96. doi: 10.1080/13674676.2020.1871328
9. Office for National Statistics. Coronavirus and the social impacts on different ethnic groups in the UK: 2020 2020 [Available from: https://www.ons.gov.uk/peoplepopulationandcommunity/culturalidentity/ethnicity/articles/coronavirusandthesocialimpactsondifferentethnicgroupsintheuk/2020 accessed March 2021.
10. Holden K, McGregor B, Thandi P, et al. Toward culturally centered integrative care for addressing mental health disparities among ethnic minorities. *Psychol Serv* 2014;11(4):357-68. doi: 10.1037/a0038122
11. Smith K, Bhui K, Cipriani A. COVID-19, mental health and ethnic minorities. *Evid Based Ment Health* 2020;23(3):89-90. doi: 10.1136/ebmental-2020-300174
12. Office for National Statistics. Coronavirus and the social impacts on Great Britain 2021 [Available from: https://www.ons.gov.uk/peoplepopulationandcommunity/healthandsocialcare/healthandwellbeing/bulletins/coronavirusandthesocialimpactsongreatbritain/29january2021 accessed March 2021.
13. Robinson E, Jones A, Lesser I, et al. International estimates of intended uptake and refusal of COVID-19 vaccines: A rapid systematic review and meta-analysis of large nationally representative samples. *MedRxiv* [Preprint]. 2020. (accessed March 2021).
14. Royal Society for Public Health. New poll finds ethnic minority groups less likely to want COVID vaccine 2020 [Available from: https://www.rsph.org.uk/about-us/news/new-poll-finds-bame-groups-less-likely-to-want-covid-vaccine.html accessed March 2021.
15. Lamb D, Gnanapragasam S, Greenberg N, et al. The psychosocial impact of the COVID-19 pandemic on 4,378 UK healthcare workers and ancillary staff: Initial baseline data from a cohort study collected during the first wave of the pandemic. *MedRxiv* [Preprint]. 2021.

For peer review only - http://bmjopen.bmj.com/site/about/guidelines.xhtml

16. Wise J. Covid-19: Study to assess pandemic's effects on wellbeing of NHS staff. *BMJ* 2020;371:m3942. doi: 10.1136/bmj.m3942
17. NHS England. North West London integrated care system (ICS) NHS England and NHS Improvement coronavirus. 2021 [March 2021]. Available from: https://www.england.nhs.uk/coronavirus/workforce/addressing-impact-of-covid-19-on-bame-staff-in-the-nhs/safe-working-for-all-nhs-staff/north-west-london-integrated-care-system-ics/.
18. Office for National Statistics. Ethnic group, national identity and religion 2011 [Available from: https://www.ons.gov.uk/methodology/classificationsandstandards/measuringequality/ethnicgroupnationalidentityandreligion accessed March 2021.
19. Richard B, Sivo SA, Orlowski M, et al. Qualitative Research via Focus Groups: Will Going Online Affect the Diversity of Your Findings? . *Cornell Hospitality Quarterly* 2021;62(1):32-45.
20. King N. Using templates in the thematic analysis of text. In: Symon G, Cassell C, eds. Qualitative Organisational Research: Core Methods and Current Challenges. London: Sage 2012:246-450.
21. Koelsch LE. Reconceptualising the member check interview. *International Journal of Qualitative Methods* 2013;12(1):168-79.
22. Public Health England. Disparities in the risk and outcomes of COVID-19: PHE Publications, 2020.
23. Willan J, King AJ, Jeffery K, et al. Challenges for NHS hospitals during covid-19 epidemic. *BMJ* 2020;368:m1117. doi: 10.1136/bmj.m1117
24. Charlesworth A. Staff shortages left the NHS vulnerable to the COVID-19 storm. The Health Foundation. 2021 [May 2021]. Available from: https://www.health.org.uk/news-and-comment/blogs/staff-shortages-left-the-nhs-vulnerable-to-the-covid-19-storm.
25. Adams T. ‘Now is the time’: London’s Black Lives Matter rally looks like a turning point. *The Guardian* 7 June 2020. https://www.theguardian.com/us-news/2020/jun/06/now-is-the-time-londons-black-lives-matter-rally-looks-like-a-turning-point (accessed March 2021).
26. Pym H. NHS: How unhappy is the public. *BBC News* 28 February 2018. https://www.bbc.com/news/health-43226197 (accessed March 2021).
27. Li J-B, Yang A, Dou K, et al. Self-control moderates the association between perceived severity of coronavirus disease 2019 (COVID-19) and mental health problems among the Chinese public. *Int J Environ Res Public Health* 2020
28. Razvi S, Oliver R, Moore J, et al. Exposure of hospital healthcare workers to the novel coronavirus (SARS-CoV-2). *Clin Med* 2020;20(6):e238-e40. doi: 10.7861/clinmed.2020-0566
29. Yildirim M, Arslan G, Ozaslan A. Perceived Risk and Mental Health Problems among Healthcare Professionals during COVID-19 Pandemic: Exploring the Mediating Effects of Resilience and Coronavirus Fear. *Int J Ment Health Addict* 2020:1-11. doi: 10.1007/s11469-020-00424-8
30. Tracy DK, Tarn M, Eldridge R, et al. What should be done to support the mental health of healthcare staff treating COVID-19 patients? *Br J Psychiatry* 2020;217(4):537-39. doi: 10.1192/bjp.2020.109
31. Shah N, Ahmed IM, Nazir T. Torn Between Caution and Compassion: a Dilemma for Clinicians from Black and Minority Ethnic Groups During the COVID-19 Pandemic. *J Racial Ethn Health Disparities* 2021;8(1):21-23. doi: 10.1007/s40615-020-00938-w

For peer review only - http://bmjopen.bmj.com/site/about/guidelines.xhtml

32. Brewin CR, Fuchkan N, Huntley Z, et al. Outreach and screening following the 2005 London bombings: usage and outcomes. *Psychol Med* 2010;40(12):2049-57. doi: 10.1017/S0033291710000206
33. Brooks SK, Dunn R, Amlot R, et al. A Systematic, Thematic Review of Social and Occupational Factors Associated With Psychological Outcomes in Healthcare Employees During an Infectious Disease Outbreak. *J Occup Environ Med* 2018;60(3):248-57. doi: 10.1097/JOM.0000000000001235
34. Milligan-Saville JS, Tan L, Gayed A, et al. Workplace mental health training for managers and its effect on sick leave in employees: a cluster randomised controlled trial. *The Lancet Psychiatry* 2017;4(11):850-58. doi: 10.1016/s2215-0366(17)30372-3
35. Naqvi H, Wilhelm R. NHS Workforce race equality standard. 2017 data analysis report for NHS trusts 2017 [Available from: https://www.england.nhs.uk/publication/workforce-race-equality-standard-data-reporting-december-2017/ accessed March 2021.
36. Otu A, Ahinkorah BO, Ameyaw EK, et al. One country, two crises: what Covid-19 reveals about health inequalities among BAME communities in the United Kingdom and the sustainability of its health system? *Int J Equity Health* 2020;19(1):189. doi: 10.1186/s12939-020-01307-z
37. Laurencin CT, Walker JM. A Pandemic on a Pandemic: Racism and COVID-19 in Blacks. *Cell Syst* 2020;11(1):9-10. doi: 10.1016/j.cels.2020.07.002
38. Lock S, Rubin GJ, Murray V, et al. Secondary stressors and extreme events and disasters: a systematic review of primary research from 2010-2011. *PLoS Curr* 2012;4 doi: 10.1371/currents.dis.a9b76fed1b2dd5c5bfcfc13c87a2f24f
39. Tempest EL, Carter B, Beck CR, et al. Secondary stressors are associated with probable psychological morbidity after flooding: A cross-sectional analysis. *Eur J Public Health* 2017;27(6):1042-7. doi: doi:10.1093/eurpub/ckx182
40. Rana W, Mukhtar S, Mukhtar S. Mental health of medical workers in Pakistan during the pandemic COVID-19 outbreak. *Asian J Psychiatr* 2020;51:102080. doi: 10.1016/j.ajp.2020.102080
41. McCanlies EC, Gu JK, Andrew ME, et al. The effect of social support, gratitude, resilience and satisfaction with life on depressive symptoms among police officers following Hurricane Katrina. *Int J Soc Psychiatry* 2018;64(1):63-72. doi: 10.1177/0020764017746197
42. Royal College of Nursing. RCN London Research Into BAME members 2018 [Available from: https://www.rcn.org.uk/news-and-events/news/rcn-london-research-into-bame-numbers accessed March 2021.
43. Gamio L. Coronavirus worker risk. *New York Times* 15 March 2020. https://www.nytimes.com/interactive/2020/03/15/business/economy/coronavirus-worker-risk.html (accessed March 2021).
44. Tan BYQ, Chew NWS, Lee GKH, et al. Psychological Impact of the COVID-19 Pandemic on Health Care Workers in Singapore. *Ann Int Med* 2020;173(4):317-20. doi: doi:10.7326/M20-1083
45. Kapilashrami A, Bhui K. Mental health and COVID-19: is the virus racist? *Br J Psychiatry* 2020;217(2):405-07. doi: 10.1192/bjp.2020.93
46. Phiri P, Delanerolle G, Al-Sudani A, et al. COVID-19 and Black, Asian, and Minority Ethnic Communities: A Complex Relationship Without Just Cause. *JMIR Public Health Surveill* 2021;7(2):e22581. doi: 10.2196/22581
47. Pendleton J. The experiences of black and minority ethnic nurses working in the UK. *Br J Nurs* 2017;26(1):37-42.

For peer review only - http://bmjopen.bmj.com/site/about/guidelines.xhtml

48. Bailey S, West M. Ethnic minority deaths and Covid-19: what are we to do? The King's Fund Blog2020 [Available from: https://www.kingsfund.org.uk/blog/2020/04/ethnic-minority-deaths-covid-19 accessed March 2021.
49. Van Kerckem K. Get under their skin and in their shoes: how to address the "Race-of-interviewer effect" in qualitative research with ethnic minorities. 9th Annual conference of the European Sociological association, Abstracts, 2009.
50. White A, Strezhnev A, Lucas C, et al. Investigator Characteristics and Respondent Behavior in Online Surveys. *J Exp Polit Sci* 2018;5(1):56-67. doi: http://dx.doi.org/10.1017/XPS.2017.25

For peer review only

For peer review only - http://bmjopen.bmj.com/site/about/guidelines.xhtml

Figure 1: Themes and subthemes identified through thematic analysis

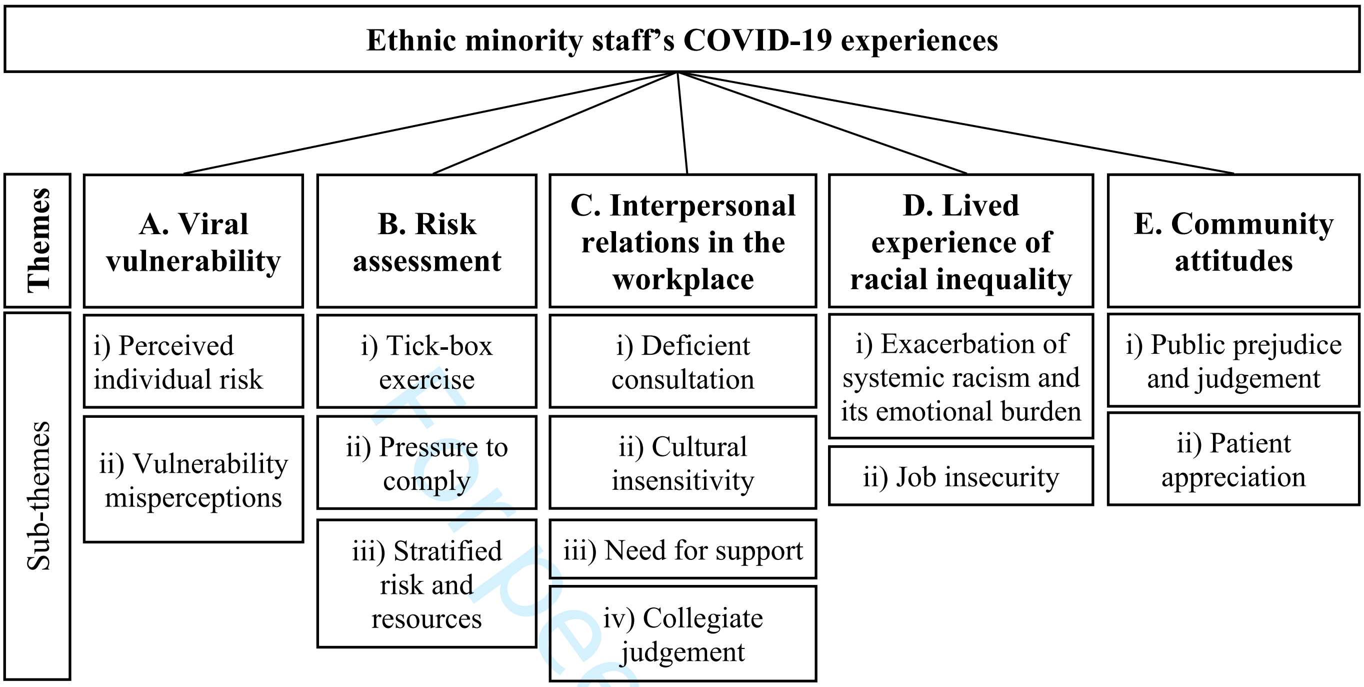

Focus group question guide

General

1. In general terms, how do you think being from BAME backgrounds has impacted upon your experiences during COVID?

Inside work experiences

2. Thinking of your experiences in your workplace, during COVID have you felt that you were treated differently from non-BAME staff? Is that by management and / or fellow workers? Why do you think that? Can you provide examples?

3. How do you think that your background has influenced your interactions with other staff members? What form has that taken? Why do you think that?

4. Did the way you were treated by your colleagues change during COVID compared to "normal times" (i.e., pre-COVID times)? If yes, how did it change? Can you give examples?

5. Did you notice any differences in the experiences of BAME and non-BAME staff during COVID? If yes, what form did they take?

6. Do you feel that you were given tasks/responsibilities that put you more at risk of contracting COVID than your non-BAME colleagues? If so, how did you respond in these situations? What was the subsequent response of the line manager requesting these tasks?

7. Do you feel others are responding to an aspect of your background in your dealings with them? If yes, what aspect do you think that is? (e.g., your ethnic group, physical appearance, culture, faith, etc).

8. Do you feel that your BAME background has affected the way you are treated by patients during COVID? If yes, please explain.

9. How well did you feel supported by your colleagues generally during COVID? If supported, how? If not supported, what support was absent and why do you think that was the case?

Outside work experiences

10. How has your home life changed during COVID? If yes, how exactly?

11. Has this affected your work life? If yes, how exactly?

12. Has there been an impact upon your children and other family members arising from their BAME background? If yes, please explain.

13. Have you experienced any changes in public life (e.g., on public transport) that you think is linked to your BAME background? If yes, please explain.

14. Has your work life affected your home life in any way? Please explain how.

For peer review only

15. If asking other BAME colleagues about their COVID experiences, what are the important questions to ask them?

16. Is there anything we didn't talk about which you think it would be useful to know?

For peer review only

MeCareNWL survey module on the experiences of healthcare staff from minority ethnic backgrounds

Q1 Do you feel your employer acknowledged BAME staff's increased vulnerability to COVID-19?

- ◯ Yes
- ◯ No
- ◯ Don't know

Q1a To what extent do you feel your employer has acknowledged BAME staff's increased vulnerability to COVID-19?

	No acknowledgement	Fully acknowledged vulnerability
	0 10 20 30 40 50 60 70 80 90 100	
1.	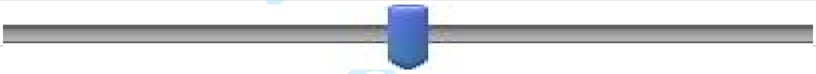	

Q2 Do you feel your employer has accommodated BAME staff's increased risk from COVID-19?

- ◯ Yes
- ◯ No
- ◯ Don't know

For peer review only

Q2b What do you feel your employer **could have done** to accommodate BAME staff's increased risk from COVID-19?

- ◯ 1. ______________________________
- ◯ 2. ______________________________
- ◯ 3. ______________________________
- ◯ 4. ______________________________
- ◯ 5. ______________________________
- ◯ 6. ______________________________
- ◯ 7. ______________________________

Q3 Did your employer take actions that made you feel safer in terms of your vulnerability to the virus?

- ◯ Yes
- ◯ No
- ◯ Not sure

Q3a What actions did your employer take that made you feel safer in terms of your vulnerability to the virus? (please select all that apply)

- ☐ Allowed me to work from home
- ☐ Provided me with adequate PPE
- ☐ Moved me to work in lower-risk wards / away from the front line
- ☐ Other (please state) ______________________________

For peer review only

Q4 Do you feel able to speak to your manager about concerns regarding your heightened vulnerability to COVID-19 due to your ethnicity?

- ◯ Yes
- ◯ No
- ◯ Don't know

Q4a To what extent do you feel able to speak to your manager about concerns regarding your vulnerability to COVID-19?

Unable to voice my concerns — Comfortable and able to speak freely

0 10 20 30 40 50 60 70 80 90 100

1.

Q5 Did you report any concerns related to your health vulnerability associated with your ethnicity to your managers?

- ◯ Yes
- ◯ No
- ◯ Don't know

Q5a To what extent did you feel that your manager understood your concerns related to your health vulnerability associated with your ethnicity?

No understanding at all — Full understanding

0 10 20 30 40 50 60 70 80 90 100

1.

For peer review only

Q6 If you worked from home during COVID-19, do you feel that your BAME status affected how much your manager trusted you while you were working from home?

- ◯ Yes
- ◯ No
- ◯ Don't know
- ◯ Didn't work from home

Q6a To what extent do you feel that your manager's trust of you while working from home is due to your ethnicity?

Less trusting Because I'm BAME — More trusting because I'm BAME

0 10 20 30 40 50 60 70 80 90 100

1.

Q7 Do you feel that you have had to take risks greater than you are comfortable with?

- ◯ Yes
- ◯ No
- ◯ Don't know

Q7a How frequently did you feel you had to take risks greater than you were comfortable with in your workplace?

Never — Very frequently

0 10 20 30 40 50 60 70 80 90 100

1.

For peer review only

Q8 Did BAME staff have to work in high-risk areas in your Trust / local authority / department / practice?

◯ Yes

◯ No

◯ Don't know

Q8a How common do you feel that the experience of BAME staff working in high-risk areas was in your Trust / local authority / department / practice?

Very rare — Very common

0 10 20 30 40 50 60 70 80 90 100

1.

Q9 Do you feel that your ethnic background played a role in your ability to obtain PPE?

◯ Yes

◯ No

◯ Don't know

Q9a To what extent do you feel that your ethnic background has played a role in your ability to obtain PPE?

Made it harder to obtain PPE — Made it easier to obtain PPE

0 10 20 30 40 50 60 70 80 90 100

1.

For peer review only

Q10 Within your workplace, which of the following provided you with a source of support when needed? (please select all that apply)

- ☐ Manager
- ☐ BAME colleagues
- ☐ Non-BAME colleagues
- ☐ Trust staff support service
- ☐ No source of support
- ☐ Other (please specify) ________________

Q11 Did you feel that patients' level of appreciation for you and other BAME staff was different from their level of appreciation for non-BAME staff?

- ○ Yes
- ○ No
- ○ Don't know

Q11a How do you feel that patient's level of appreciation for staff differed between BAME staff and non-BAME staff?

Less appreciation for BAME staff than non-BAME staff	More appreciation for BAME staff than non-BAME staff

0 10 20 30 40 50 60 70 80 90 100

1. 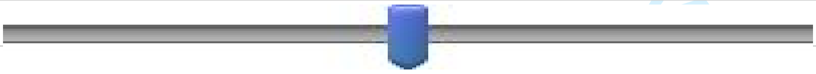

Q12 Did you **expect** to experience increased prejudice associated with your ethnic background as a result of COVID-19 **in your workplace**?

- ○ Yes
- ○ No
- ○ Don't know

For peer review only

Q13 Did you **actually** experience increased prejudice associated with your ethnic background as a result of COVID-19 **in your workplace**?

- ◯ Yes
- ◯ No
- ◯ Don't know

Q14 Did you **expect** to experience increased prejudice associated with your ethnic background as a result of COVID-19 **in the community**?

- ◯ Yes
- ◯ No
- ◯ Don't know

Q15 Did you **actually** experience increased prejudice associated with your ethnic background as a result of COVID-19 **in the community**?

- ◯ Yes
- ◯ No
- ◯ Don't know

Q16 What practical suggestions do you have that would help you feel **heard** by management?

____

Q17 What practical suggestions do you have that would help you feel **safer** in your workplace?

____

For peer review only

COREQ (COnsolidated criteria for REporting Qualitative research) Checklist

A checklist of items that should be included in reports of qualitative research. You must report the page number in your manuscript where you consider each of the items listed in this checklist. If you have not included this information, either revise your manuscript accordingly before submitting or note N/A.

Topic	Item No.	Guide Questions/Description	Reported on Page No.
**Domain 1: Research team and reflexivity**			
Personal characteristics			
Interviewer/facilitator	1	Which author/s conducted the interview or focus group?	
Credentials	2	What were the researcher's credentials? E.g. PhD, MD	
Occupation	3	What was their occupation at the time of the study?	
Gender	4	Was the researcher male or female?	
Experience and training	5	What experience or training did the researcher have?	
Relationship with participants			
Relationship established	6	Was a relationship established prior to study commencement?	
Participant knowledge of the interviewer	7	What did the participants know about the researcher? e.g. personal goals, reasons for doing the research	
Interviewer characteristics	8	What characteristics were reported about the inter viewer/facilitator? e.g. Bias, assumptions, reasons and interests in the research topic	
**Domain 2: Study design**			
Theoretical framework			
Methodological orientation and Theory	9	What methodological orientation was stated to underpin the study? e.g. grounded theory, discourse analysis, ethnography, phenomenology, content analysis	
Participant selection			
Sampling	10	How were participants selected? e.g. purposive, convenience, consecutive, snowball	
Method of approach	11	How were participants approached? e.g. face-to-face, telephone, mail, email	
Sample size	12	How many participants were in the study?	
Non-participation	13	How many people refused to participate or dropped out? Reasons?	
Setting			
Setting of data collection	14	Where was the data collected? e.g. home, clinic, workplace	
Presence of non-participants	15	Was anyone else present besides the participants and researchers?	
Description of sample	16	What are the important characteristics of the sample? e.g. demographic data, date	
Data collection			
Interview guide	17	Were questions, prompts, guides provided by the authors? Was it pilot tested?	
Repeat interviews	18	Were repeat inter views carried out? If yes, how many?	
Audio/visual recording	19	Did the research use audio or visual recording to collect the data?	
Field notes	20	Were field notes made during and/or after the inter view or focus group?	
Duration	21	What was the duration of the inter views or focus group?	
Data saturation	22	Was data saturation discussed?	
Transcripts returned	23	Were transcripts returned to participants for comment and/or	

For peer review only

Topic	Item No.	Guide Questions/Description	Reported on Page No.
		correction?	
**Domain 3: analysis and findings**			
Data analysis			
Number of data coders	24	How many data coders coded the data?	
Description of the coding tree	25	Did authors provide a description of the coding tree?	
Derivation of themes	26	Were themes identified in advance or derived from the data?	
Software	27	What software, if applicable, was used to manage the data?	
Participant checking	28	Did participants provide feedback on the findings?	
Reporting			
Quotations presented	29	Were participant quotations presented to illustrate the themes/findings? Was each quotation identified? e.g. participant number	
Data and findings consistent	30	Was there consistency between the data presented and the findings?	
Clarity of major themes	31	Were major themes clearly presented in the findings?	
Clarity of minor themes	32	Is there a description of diverse cases or discussion of minor themes?	

Developed from: Tong A, Sainsbury P, Craig J. Consolidated criteria for reporting qualitative research (COREQ): a 32-item checklist for interviews and focus groups. *International Journal for Quality in Health Care*. 2007. Volume 19, Number 6: pp. 349 – 357

Once you have completed this checklist, please save a copy and upload it as part of your submission. DO NOT include this checklist as part of the main manuscript document. It must be uploaded as a separate file.

For peer review only

BMJ Open

“We weren't checked in on, nobody spoke to us”: An exploratory qualitative analysis of ethnic minority NHS staff concerns during COVID-19

Journal:	*BMJ Open*
Manuscript ID	bmjopen-2021-053396.R1
Article Type:	Original research
Date Submitted by the Author:	28-Sep-2021
Complete List of Authors:	Jesuthasan, Jehanita; Imperial College London Faculty of Medicine, Department of Brain Sciences<br>Powell, Richard A; Imperial College London School of Public Health, Department of Primary Care and Public Health; NIHR Collaboration for Leadership in Applied Health Research and Care for Northwest London<br>Burmester, Victoria; Imperial College London Faculty of Medicine, Department of Brain Sciences<br>Nicholls, Dasha; Imperial College London Faculty of Medicine, Department of Brain Sciences; NIHR Collaboration for Leadership in Applied Health Research and Care for Northwest London
**Primary Subject Heading**:	Mental health
Secondary Subject Heading:	Health services research, Qualitative research
Keywords:	COVID-19, MENTAL HEALTH, Health & safety < HEALTH SERVICES ADMINISTRATION & MANAGEMENT, OCCUPATIONAL & INDUSTRIAL MEDICINE

SCHOLARONE™
Manuscripts

I, the Submitting Author has the right to grant and does grant on behalf of all authors of the Work (as defined in the below author licence), an exclusive licence and/or a non-exclusive licence for contributions from authors who are: i) UK Crown employees; ii) where BMJ has agreed a CC-BY licence shall apply, and/or iii) in accordance with the terms applicable for US Federal Government officers or employees acting as part of their official duties; on a worldwide, perpetual, irrevocable, royalty-free basis to BMJ Publishing Group Ltd ("BMJ") its licensees and where the relevant Journal is co-owned by BMJ to the co-owners of the Journal, to publish the Work in this journal and any other BMJ products and to exploit all rights, as set out in our licence.

The Submitting Author accepts and understands that any supply made under these terms is made by BMJ to the Submitting Author unless you are acting as an employee on behalf of your employer or a postgraduate student of an affiliated institution which is paying any applicable article publishing charge ("APC") for Open Access articles. Where the Submitting Author wishes to make the Work available on an Open Access basis (and intends to pay the relevant APC), the terms of reuse of such Open Access shall be governed by a Creative Commons licence – details of these licences and which Creative Commons licence will apply to this Work are set out in our licence referred to above.

Other than as permitted in any relevant BMJ Author's Self Archiving Policies, I confirm this Work has not been accepted for publication elsewhere, is not being considered for publication elsewhere and does not duplicate material already published. I confirm all authors consent to publication of this Work and authorise the granting of this licence.

For peer review only

"We weren't checked in on, nobody spoke to us": An exploratory qualitative analysis of ethnic minority NHS staff concerns during COVID-19

Jehanita Jesuthasan[1]
Richard A Powell[2, 3]
Victoria ML Burmester[1]
Dasha Nicholls[1, 3]

[1] Imperial College London Faculty of Medicine, Department of Brain Sciences, London, UK
[2] Imperial College London School of Public Health, Department of Primary Care and Public Health, London, UK
[3] National Institute for Health Research Collaboration for Leadership in Applied Health Research and Care for Northwest London, London, UK

For peer review only

Correspondence to:
Dr Dasha Nicholls
Mailing address: Department of Brain Sciences, Faculty of Medicine, Imperial College London, Floor 2, Commonwealth Building The Hammersmith Hospital, Du Cane Road, London W12 0NN
Email: d.nicholls@imperial.ac.uk
Tel: +44 (0)20 3313 8436

Word count: 4250

For peer review only - http://bmjopen.bmj.com/site/about/guidelines.xhtml

Statement of ethics approval: The MeCare study received ethical approval from the Health Research Authority (IRAS Project ID 290383), which included permission to co-create a module addressing questions specifically for ethnic minority populations with input from Patient and Public Involvement (PPI) colleagues, project partners and other experts by experience. Participants gave their informed consent before taking part.

Funding: This report is independent research funded by the National Institute for Health Research North West London Applied Research Collaboration (ARC) and by the Imperial College COVID-19 Research Fund grant number P88408. The views expressed in this publication are those of the authors and not necessarily those of the National Institute for Health Research or the Department of Health and Social Care.

Competing interests: None declared.

Data sharing statement: No additional data are available.

Author contributions: JJ, RAP, VB, and DN conceptualised the study. RAP conducted the focus groups. JJ, RAP, and VB analysed the data. JJ wrote the draft with support from RAP, VB, and DN. RAP, VB, and DN managed the overall design of the study.

Non-author contributors: John Norton and Sandra Jayacodi, partners of the National Institute for Health Research ARC Northwest London PPI Initiative, verified data interpretation and reviewed the final manuscript.

Acknowledgements: We would like to thank the participants for taking part in this research and sharing their experiences.

Figures

Figure 1: Thematic Experiences of Ethnic Minority Healthcare Staff During COVID-19

Supplementary Materials

For peer review only - http://bmjopen.bmj.com/site/about/guidelines.xhtml

1. Interview guide for the focus groups

2. Survey module created based on the focus groups

For peer review only

For peer review only - http://bmjopen.bmj.com/site/about/guidelines.xhtml

ABSTRACT

Objective

To examine the multi-faceted, lived-experience impact of COVID-19 on ethnic minority healthcare staff to co-create a module of questions for follow-up online surveys on the wellbeing of healthcare staff during the pandemic.

Design

A cross-sectional design using two online focus groups among ethnic minority healthcare workers who worked in care or supportive roles in a hospital, community health or GP surgery setting for at least 12 months.

Participants

Thirteen healthcare workers (11 female) aged 26 to 62 years from diverse ethnic minority backgrounds, ten working in clinical roles.

Results

Five primary thematic domains emerged: 1) *viral vulnerability*, centring around perceived individual risk and vulnerability perceptions; 2) *risk assessment*, comprising of pressures to comply, perception of a tick-box exercise, and issues with risk and resource stratification; 3) *interpersonal relations in the workplace*, highlighting deficient consultation of ethnic minority staff, cultural insensitivity, need for support, and collegiate judgement; 4) *lived experience of racial inequality*, consisting of job insecurity and the exacerbation of systemic racism and its emotional burden; 5) *community attitudes*, including public prejudice and judgement, and patient appreciation.

For peer review only

For peer review only - http://bmjopen.bmj.com/site/about/guidelines.xhtml

Conclusions

Our novel study has shown ethnic minority NHS staff have experienced COVID-19 in a complex, multi-dimensional manner. Future research should enumerate the extent to which these varied thematic experiences are shared among ethnic minority NHS workers so that more empathetic and supportive management and related occupational practices can be instituted.

ARTICLE SUMMARY

Strengths and limitations of this study

- This is one of the first studies to examine qualitatively the experiences of ethnic minority healthcare staff during the COVID-19 pandemic.
- Due to the self-selected nature of the sample, the experiences of participants may not be representative of the ethnic minority healthcare workforce as a whole.
- Female healthcare workers were disproportionately represented in our sample.
- Given its exploratory nature, the sample size was potentially insufficient to achieve data saturation.

KEYWORDS: COVID-19; Mental Health; Occupational Health; Health and Safety

For peer review only

For peer review only - http://bmjopen.bmj.com/site/about/guidelines.xhtml

INTRODUCTION

COVID-19 has adversely impacted the occupational roles and physical and mental wellbeing of healthcare staff[1]. Many have experienced disrupted support structures, redeployment to areas outside their professional training[2]—sometimes engaging in tasks that transgress individual moral consciences and values[3]—limited resources and vital medical equipment[4], and unprecedented patient service demand amid rapidly changing care guidelines[5]. Staff are not only vulnerable to contracting the virus but at risk of work stress, moral injury, and mental ill-health, including clinical depression, post-traumatic stress disorder, substance misuse, and suicide[6].

Ethnic minority care staff have been disproportionately affected by the pandemic's clinical impact[7].The impact is multi-faceted: economic, social, attitudinal and cultural, as well as occupational, physical and psychological[8 9]. Understanding these broader impacts can facilitate a more holistic and empathetic understanding of the lived experiences of COVID-19 among health care staff from ethnic minorities, generating greater awareness of health-seeking behaviour in and outside the workplace, and perceived impediments to seeking and receiving support[10 11]. Increased understanding can also inform public health campaigns addressing, for example, disinclination to accept vaccines[12-14].

The MeCare study in Northwest (NW) London, collaborating with partners in the larger *NHS Check* study[15 16], is longitudinally examining the mental health and wellbeing of National Health Service (NHS) staff (including general practitioners) in NW London—where 49% of NHS staff are from ethnic minority backgrounds[17]—and exploring how to support staff during pandemics. This paper reports on the co-creation of a module of questions targeted towards staff from ethnic minority backgrounds as part of follow-up online surveys.

For peer review only - http://bmjopen.bmj.com/site/about/guidelines.xhtml

METHOD

Participants

We explored the experiences of ethnic minority healthcare workers selected through purposive sampling. Participants were recruited through Patient and Public Involvement (PPI) networks and promotional materials circulated among NHS trusts. Participants were adults from an ONS[18] ethnic minority background who worked in care or supportive roles in a hospital, community health or GP surgery setting for at least 12 months.

Before beginning, the purpose of the focus group, the purpose of recording the sessions, the anonymisation of responses for analysis and publication were reiterated. Participants were made aware that the sessions were to be recorded to facilitate subsequent analysis and informed that by attending they were agreeing to the recording of their contribution. The recordings were only to be available to the research team and were to be destroyed after they had been transcribed. Participants were also reminded that they could withdraw their participation at any time from the sessions with no consequences but that their responses up to that withdrawal point would be retained. No participants withdrew from the online meeting or afterwards; however, two participants decided not to participate on the day of the sessions: one because of IT connectivity challenges, the other because of a work emergency. Participants were offered the opportunity to ask any questions before providing their informed consent orally before taking part.

Data collection

Two 2-hour focus groups of 6 and 7 participants were held online—shown to generate idea diversity comparable to in-person equivalents[19]—using Microsoft Teams (MT) and facilitated by RAP, with participants offered the choice of having their session cameras live or not. VB was present at the beginning of both sessions to introduce the research and JJ was

For peer review only - http://bmjopen.bmj.com/site/about/guidelines.xhtml

present as a note-taker throughout both sessions. Field notes were taken by JJ on key points and the conversations were recorded using MT's software, from which manuscripts were produced. Both groups followed the study interview guide (Supplementary File 1), which was reactively adapted, in terms of questioning order and prompts. The interview guide was developed based on existing research and news reports on the experiences of ethnic minority healthcare staff in the UK during the pandemic, and upon guidance from an expert team at King's College London and independently reviewed and refined by RP, VB and JJ. In conducting the group discussions, RAP ensured each participant was able to understand and respond to each question, if they so wished. Participants also had the opportunity to respond to each other's comments and responses.

Data analysis

Discussions were transcribed verbatim and managed using MTs' automatic caption generation software and manually checked for errors. RAP, VB and JJ undertook independent content analysis using the template analysis approach[20], under a constructivist qualitative research paradigm, combining deductive and inductive analyses. The study team subsequently met to reach consensus on emergent themes and sub-themes. To ensure the veracity of data interpretation, the findings and draft survey questions were validated by member checking with a sample of group participants[21].

Patient and public involvement

Participants were recruited through PPI representatives and derived separately from the MeCare study's target population, they were not direct study participants. JN and SJ are public partners of the Applied Research Collaboration in NW London and provided feedback on data interpretation.

For peer review only - http://bmjopen.bmj.com/site/about/guidelines.xhtml

RESULTS

The final sample included 13 healthcare workers (11 female) with a mean age of 42.7 years (SD = 10.3 years). Ethnically the sample was comprised of: Eastern European (n = 1), Indian (n = 2), Mixed White and Black Caribbean (n = 1), Caribbean (n = 1), Mixed White and Asian (n = 1), African (n = 2), Arab (n = 1), Bangladeshi (n = 1), Asian Other (n = 2), and Other, in this instance Mixed North African and Eastern European, (n = 1). Eleven worked in clinical roles in primary and secondary care settings (one GP, three consultants, one nurse, one clinical lead at an urgent care team, two hospital doctors, one pharmacist, one patient housing officer, and one psychological wellbeing practitioner); two worked in administrative roles (one hospital administrator and one assistant improvement manager).

The five primary thematic domains that emerged were: viral vulnerability, risk assessment, interpersonal relationships in the workplace, lived experience of racial inequality, and community attitudes (Figure 1).

Figure 1: Themes and sub-themes identified through thematic analysis.

1) Viral vulnerability

a) Perceived individual risk

Participants were sensitive to their increased risk of infection compared to non-ethnic minority colleagues in the same roles. They discussed the impact of this heightened risk on their emotional wellbeing, reporting personal anxiety and emotional distress.

For peer review only - http://bmjopen.bmj.com/site/about/guidelines.xhtml

> P12: "I think most upsetting was the initial kind of response and risk stratifications and the deaths of people I knew or knew by indirect means."

Participants also expressed frustration that individual vulnerability was insufficiently acknowledged within their teams and organisations, a shortcoming one participant suggested was associated with the fact that it was ethnic minorities rather than the non-minority population who were affected.

> P2: "If the prevalence and incidence was affecting the White majority population more than the Black population, maybe something could be done differently."

b) Vulnerability misperceptions

Several participants expressed frustration about fundamental misunderstandings regarding the reasons for increased infection risk among ethnic minorities: in public discourse and government reports, differences in viral vulnerability were frequently attributed to individual factors (personal hygiene practices, individual obesity). Participants felt insufficient attention was paid to structural determinants, from exposure differentials to unconscious racism.

> P3: "That research was basically saying that [ethnic minorities] are at more risk because they are obese, they have got Type 2 diabetes, they're living in

For peer review only - http://bmjopen.bmj.com/site/about/guidelines.xhtml

> overcrowded situations and all that. […] They did not consider that this could be just because we are exposed more."

> P13: "It's just being aware that it's not entirely biological, the risk that they face. Some of the reasons why we saw people from BAME [Black, Asian and minority ethnic] background suffer a lot more was because of their social circumstances as well."

For peer review only

2) Risk assessment

a) Tick-box exercise

Many participants felt risk assessment exercises had not been treated seriously by their managers and organisations. Although some felt their employer accommodated their identified increased risk, many reported not seeing tangible protective actions for vulnerable ethnic minority staff. By not emphasising the importance of completing risk assessments, and taking minimal action following them, management made staff feel the assessment was a tick-box exercise rather than a considered strategy to reduce staff risk.

> P3: "I wasn't able to understand what the point of that risk assessment was and if somebody was found to be high risk population, whether something different was done for that person."

For peer review only - http://bmjopen.bmj.com/site/about/guidelines.xhtml

b) Pressure to comply

Despite awareness of ethnic minority communities' COVID-19 vulnerability, participants reported feeling under pressure to continue frontline working. This arose both from managers and an innate sense of duty, with a sense of guilt associated with not seeing in-person patients among higher-risk staff members who were shielding and had moved to remote working.

> P11: "I did feel quite guilty about not being at work seeing patients. […] Every Thursday for a while [my neighbours] were all standing outside clapping and I didn't go outside. I stayed inside and hid because I felt like a complete fraud."

Other participants who were still working in person frequently felt they had little choice but to continue working in high-risk positions.

> P5: "You kind of feel you're obligated to follow through your duties, but there still is that major risk of putting yourself or your health at risk."

In terms of extrinsic pressure, the demands on NHS frontline staff limited the alternatives to positioning ethnic minority staff in frontline care provision. Consequently, many reported no choice but to maintain their position on high-risk wards—even when Personal Protective Equipment (PPE) was in short supply—and being pressured to return to work shortly after COVID-19 diagnoses.

For peer review only - http://bmjopen.bmj.com/site/about/guidelines.xhtml

P5: "If you pull out all the people on the frontline, then who's going to actually do the work? Who's gonna fill in those gaps?"

P12: "They were desperate to get people on the ground and there was this whole thing about 'it's a respiratory virus and it only lasts 2 weeks max'. So, many members of staff were harassed, told 'you have to go back, your two weeks are up'."

P4: "Your personal risk doesn't matter, the managers are managers, they're there to get the job done, to apply the pressure coming from above. So, if you're a nurse in a unit that is short-staffed and you turn up to work and they don't have [the necessary PPE], you either get on with your job knowing that you're at high risk, or you become a problem."

c) Stratified risk and resources

Several participants identified flaws in the risk and resultant resources stratification, feeling the organisation's workplace hierarchy was imposed on the risk assessment. They contended the distribution of PPE was based on professional hierarchy rather than individual vulnerability.

P4: "The distribution of PPE seems to have followed a hierarchical structure. So, medics are walking around with 3M rubber fit-sealed

For peer review only - http://bmjopen.bmj.com/site/about/guidelines.xhtml

> masks, although they don't spend that much time in COVID areas, while some of the Filipino nurses are struggling to find a 3M mask that fits them. And then when it comes to risk assessing the domestic staff, they're an afterthought."

3) Interpersonal relations in the workplace

a) Deficient consultation

The perception of blame for increased vulnerability to COVID-19 being ascribed to ethnic minority communities and a disregard for the role of structural factors fuelled belief in a lack of understanding of ethnic minority experiences. Moreover, the view that attempts to remedy this deficiency were lacking reinforced the sense that larger racial issues were disregarded and thereby unsolved. Participants reported ethnic minorities were excluded from conversations concerning them—such as the construction of risk assessments—and felt the impacts of their vulnerability on their wellbeing were disregarded within organisations.

> P1: "We weren't checked in on, nobody spoke to us to see if we were okay, how we felt about it. It just wasn't really made a big deal of. It wasn't something that mattered."

> P2: "The issue of not being heard, as somebody from the BAME community. 'Cause I felt like, up to now, nobody's really asked me 'What do you want? What can we do to make you feel protected?' So, I'm aware that the risk assessment was imposed by other people from

For peer review only - http://bmjopen.bmj.com/site/about/guidelines.xhtml

other organisations. […] It was again that issue of them discussing about us without us."

b) Cultural insensitivity

The disconnect between ethnic and non-ethnic minority staff was associated with difficulties sharing concerns with non-minority supervisors. Several participants cited cultural differences and the associated lack of understanding of ethnic minority staff's experiences, needs, and problems as the principal barrier to discussing concerns with non-ethnic minority managers. Indeed, it was suggested that a common background with one's supervisor would facilitate communication and increase feelings of being supported.

P4: "I was staying in the trust hotel and went out for a walk […] I was subsequently stopped and searched by police, assaulted. Trying to explain that to my manager resulted in the eye-rolling from them, and them not really understanding the consequences of that."

P3: "It is always much easier to explain to people who are from your own cultural background or somebody who has experienced the same kind of thing rather than actually going to your manager who has got no idea what you're talking about."

For peer review only - http://bmjopen.bmj.com/site/about/guidelines.xhtml

For example, the difficulty obtaining hair caps, needed by ethnic minority staff who do not wash their hair daily, highlighted the impact of under-representation of such staff in senior positions and ethnic minority staff's ability to perform their jobs.

c) Need for support

Several participants stated they did not feel adequately supported by their employer and their manager during the pandemic. Some felt particularly unsupported when their supervisor was from a non-ethnic minority background, making it difficult to know their concerns related to ethnicity were heard. For participants who felt they had organisational support, it was seen as improving their wellbeing and may have mitigated the effects of racial injustices they were increasingly aware of and subjected to during the pandemic.

> P3: "Will [ethnic minority staff] feel more comfortable and more supported if they have somebody appointed that they can approach if they have any issues? […] Will they feel more comfortable if that person is from the same cultural background?"

> P11: "I think people were quite protective of me [because I'm ethnic minority] and I personally didn't find that paternalistic or patronising. I felt cared for."

d) Collegiate judgement

Several participants were wary of special treatment being accorded to them, fearing it might negatively impact how they are viewed by non-ethnic minority colleagues. It was

For peer review only - http://bmjopen.bmj.com/site/about/guidelines.xhtml

suggested some ethnic minority staff may have been reluctant to be risk-stratified and made to work from home for fear of being negatively judged by team members.

> P11: "There were a significant minority of people who, although my recommendation was that they should work from home, they chose to not follow guidance and they wanted to see patients face to face. […] I suspect that might have been because they were worried about their position in the team and about bullying."

Participants also noted that, in some cases, the special attention given to ethnic minority individuals was experienced as discrimination and a cause for bullying.

> P11: "There were a lot of people who were BAME who did feel that there was a spotlight being shone upon them. […] A lot of them didn't feel that that was positive. They felt that it could be seen as quite discriminatory, as them being given special privileges."

Moreover, several participants were defensive of the reasons for doing their jobs, emphasising their interest in, and passion for, their work and denying ulterior motives, such as hopes to receive special treatment.

> P9: "It's work that the BAME group tends to have interest in, and we do it with a love for it, rather than wanting to be specially treated. I

For peer review only - http://bmjopen.bmj.com/site/about/guidelines.xhtml

think sometimes when there's a lot of talk about BAME groups, it can be a little bit patronising because you feel that's a career that you've always wanted to do."

This further highlights the fear of one's job performance being judged by colleagues based on ethnicity.

4) Lived experience of racial inequality

a) Exacerbation of systemic racism and its emotional burden

There was a strong sense that existing structural workplace inequality and the emotional burden of racial injustice were exacerbated during the pandemic. Participants felt ethnic minority staff have a distinct disadvantage in career advancement, noting they had to work harder than non-minority counterparts to progress their careers. Crucially, several participants felt that increased COVID-19 vulnerability compounded career advancement challenges, and that the pandemic provided an opportunity for unconscious bias among management to manifest in career advancement opportunities given to non-ethnic minority staff.

P11: "I think that there's something similar [to being female] about being BAME compared to being White, where you know you have to work so much harder to get where you want to go to. […] Then there's COVID affecting us in a more serious way, […] it's another disadvantage."

For peer review only - http://bmjopen.bmj.com/site/about/guidelines.xhtml

> P4: “There are at least a few cases of people in the same staff group occupying two bandings. And unfortunately, BAME staff at one, slightly lower, band. And management have taken it upon themselves to use the chaos that’s going on to promote the same types of people that always get promoted.”

Additionally, participants noted the emotional burden of the pandemic is especially high among ethnic minority staff and that the Black Lives Matter movement compounded feelings of racial injustice evoked in the workplace during the pandemic and highlighted structural, societal problems.

> P1: “All of the Black Lives Matter stuff that was going on, and George Floyd and the protests, the whole attitude around them in the workplace was just exacerbating people's feelings and made it very difficult. It felt like you were being attacked from all sides.”

b) Job insecurity

Participants also reported the perception that working from home may threaten their job due to racial inequality in the workplace and that managers were less trusting of ethnic minority staff working from home than non-ethnic minority counterparts.

For peer review only - http://bmjopen.bmj.com/site/about/guidelines.xhtml

P11: “I found that the drawback [of working from home] was I wasn't getting my face seen [at work]. […] I actually almost felt that my job was threatened, I became very anxious and very insecure about my place within the team.”

P9: “[At the time I was working from home] I had a colleague I was working with who felt that that pressure [from managers] was given to the ethnic minority group because we were not trusted to be doing what we should be doing.”

5) Community attitudes

a) Public prejudice and judgement

The racial injustice experienced by ethnic minority staff in their workplace also occurred in the community, where participants reported witnessing racist slurs against East Asians and anticipating an increase in such attitudes towards themselves.

P10: “I've felt a lot more worried about having sort of racial attacks or racial comments pointed in my direction, given some of the narrative around COVID, such as the ‘Chinese Virus’.”

Additionally, the ethnicity-based judgements participants reported receiving from the public reinforced their understanding that ethnic minority staff were seen as less than non-ethnic minority colleagues in society.

For peer review only - http://bmjopen.bmj.com/site/about/guidelines.xhtml

> P5: “You feel like although you're coming to do something positive but at the same time other people might be making all sorts of judgments as to what you're doing.”

> P5: “One of my neighbours said I shouldn't be going outside, so, I said ‘No, I work for the NHS’ and automatically his response was ‘Oh! Are you a cleaner?’.”

b) Patient appreciation

Despite racial prejudices experienced in the community, participants noted patients showed an increased recognition of the value ethnic minority communities bring to the NHS and felt this increased patient appreciation was reflective of a broader attitudinal shift towards their key role.

> P6: “[Patients] were very happy to be seen, happy to be taken care of, they were politer. They really appreciated what we were doing.”

> P1: “I think there was a shift in [patients’] attitude towards NHS staff in general, which was refreshing.”

In contrast, others were sceptical of whether this recognition was indicative of a real attitudinal shift, recalling past NHS criticism. Moreover, participants highlighted a disconnect

For peer review only - http://bmjopen.bmj.com/site/about/guidelines.xhtml

between the public's appreciation of the NHS, expressed in weekly street clapping, and practical acknowledgement of the risk to which NHS staff are regularly exposed.

> P13: "When people would clap for the NHS I would think 'Oh my God they're clapping. What are they talking about? Do they even know what happens inside?'."

DISCUSSION

This exploratory group process examined the lived experiences of healthcare staff from ethnic minority backgrounds during COVID-19 to develop an ethnically empathetic module for follow-up survey stages in the MeCare study (Supplementary File 2). The five primary themes that emerged from our research have highlighted key areas of concern and neglect that need to be enumerated and investigated further to appreciate the extent to which they are shared by ethnic minority staff.

These concerns are consistent with research regarding the depression, anxiety, and stress associated with perceived COVID-19 risk and the pandemic's impact on ethnic minority communities[22-24]. The perceived pressure to work despite individual risk can significantly impact staff's work performance and mental health. For example, presenteeism—individuals presenting at work but operating sub-optimally due to health issues—can have serious consequences if it leads to poor, slow, or incorrect decision-making[25]. Moreover, Shah et al.[26] reported that during the pandemic, ethnic minority staff faced dilemmas around fulfilling their duty and continuing to deliver patient clinical care or exercising mitigating actions to avoid high-risk environments. This is especially relevant given the elevated extrinsic pressure on ethnic minority NHS frontline staff amid surges in

For peer review only - http://bmjopen.bmj.com/site/about/guidelines.xhtml

COVID-19 cases[27], combined with staffing shortages[28] and the high proportion of ethnic minority NHS staff[17].

Ethnic minority staff's mental health is likely to also be impacted by a disconnect between ethnic minority and non-minority staff. Indeed, post-trauma social support from managers influences staff's long-term mental health status[29 30], and discussion of ethnic minority staff's experiences during COVID-19 with non-minority supervisors can help foster improved attitudes towards mental health in the workplace[31]. The disconnect was manifested in fear of bullying, which is consistent with evidence that ethnic minority staff are more likely than non-minority colleagues to experience staff bullying[32 33]. Additionally, participants' sense that the pandemic exacerbated existing emotional burdens regarding systemic racism is consistent with an interplay between racism and the pandemic[34]. This may also contribute to longer-term mental ill-health—including the psychological consequences of trauma exposure[29 35 36]—among ethnic minority staff[37]. On a positive note, however, the public's acknowledgement of work undertaken by staff during the pandemic helps promote staff's resilience, thereby protecting their mental health[38].

Participants' accounts also suggest a perception that nurses and domestic staff—who are more likely to be from ethnic minority backgrounds[39] and are particularly vulnerable to infection due to their physical proximity to patients when providing care[40]—struggled to secure adequate PPE, which was more easily available for senior staff. The perception that PPE distribution was based on professional hierarchy is important. Previous research has found that, among healthcare workers, the incidence of anxiety was highest among non-medical healthcare staff, possibly because they had less first-hand information about the disease and less training on infection control measures and PPE use[41]. Additionally, adequate consultation of ethnic minority staff is especially important given they are already less likely

For peer review only - http://bmjopen.bmj.com/site/about/guidelines.xhtml

than non-minority counterparts to voice their opinions and raise concerns about inadequate PPE, increasing the likelihood of their needs being overlooked[33 42].

Racial inequalities were also reported in career advancement and treatment by managers. This is consistent with reports that ethnic minority staff have been restricted to certain NHS roles due to inequalities in career development and workplace and societal discrimination, rendering them more likely to work in critical specialties and services during the pandemic[43]. Moreover, ethnic minority staff are also more likely than non-ethnic minority staff to experience excessive scrutiny and punishment[44] and discrimination[45] from their managers.

In terms of study limitations, an important limitation was that the sample was self-selected and, as such, participants were likely to feel more strongly about their experience of the pandemic compared to the ethnic minority workforce as a whole. However, participants were purposively sought for their opinions to gain broad insights into this neglected area to inform further inquiry, rather than generating representative data. The MeCare module that this research informed will provide an opportunity to collect data from a broader range of ethnic minority staff and enable exploration of whether these experiences were representative and resonate with ethnic minority staff.

Second, female healthcare workers were disproportionately represented in our sample, potentially omitting specifically male experiences during the pandemic. Third, there is the possibility that the facilitator's race (non-minority ethnic) biased the data by inhibiting open discussion[46]. However, the facilitator has over 20 years' experience of health research in diverse settings including low- and middle-income countries, and JJ, who was present in both focus groups, is minority ethnic. In addition, discussions were frank and unimpeded, and evidence indicates the impacts of the interviewer's ethnicity are minimal[47]. Fourth, the sample size was potentially insufficient to achieve thematic saturation as this was an

For peer review only - http://bmjopen.bmj.com/site/about/guidelines.xhtml

exploratory exercise to highlight largely unexamined thematic areas that are currently being further researched. To minimize the possibility of the authors' invalid interpretation of the data, the study used respondent validation among a sample of group participants.

Our novel study has clearly shown that ethnic minority NHS staff have experienced COVID-19 in a complex, multi-dimensional manner. The physical and psychological impact of the virus has been overlayed by wider occupational, attitudinal (including anticipatory racism) and socio-cultural experiences. A need exists to understand the extent to which these varied experiences are shared among ethnic minority NHS workers so more empathetic and supportive management and related occupational practices can be instituted.

For peer review only

For peer review only - http://bmjopen.bmj.com/site/about/guidelines.xhtml

References

1. Gilleen J, Santaolalla A, Valdearenas L, et al. The impact of the COVID-19 pandemic on the mental health and wellbeing of UK healthcare workers. *MedRxiv* [Preprint]. 2020. (accessed March 2021).
2. Vera San Juan N, Camilleri M, Jeans JP, et al. Redeployment and training of healthcare professionals to Intensive Care during COVID-19: a systemic review. *MedRxiv* [Preprint]. 2021. (accessed March 2021).
3. Newdick C, Sheehan M, Dunn M. Tragic choices in intensive care during the COVID-19 pandemic: on fairness, consistency and community. *J Med Ethics* 2020;46(10):646-51. doi: 10.1136/medethics-2020-106487
4. Supady A, Curtis JR, Abrams D, et al. Allocating scarce intensive care resources during the COVID-19 pandemic: practical challenges to theoretical frameworks. *The Lancet Respiratory Medicine* 2021;9(4):430-34. doi: 10.1016/s2213-2600(20)30580-4
5. Vindrola-Padros C, Andrews L, Dowrick A, et al. Perceptions and experiences of healthcare workers during the COVID-19 pandemic in the UK. *BMJ Open* 2020;10(11):e040503. doi: 10.1136/bmjopen-2020-040503
6. Greenberg N. Mental health of health-care workers in the COVID-19 era. *Nat Rev Nephrol* 2020;16(8):425-26. doi: 10.1038/s41581-020-0314-5
7. Shields A, Faustini SE, Perez-Toledo M, et al. SARS-CoV-2 seroprevalence and asymptomatic viral carriage in healthcare workers: a cross-sectional study. *Thorax* 2020;75(12):1089-94. doi: 10.1136/thoraxjnl-2020-215414
8. Jaspal R, Lopes B. Discrimination and mental health outcomes in British Black and South Asian people during the COVID-19 outbreak in the UK. *Mental Health, Religion & Culture* 2021;24(1):80-96. doi: 10.1080/13674676.2020.1871328
9. Office for National Statistics. Coronavirus and the social impacts on different ethnic groups in the UK: 2020 2020 [Available from: https://www.ons.gov.uk/peoplepopulationandcommunity/culturalidentity/ethnicity/articles/coronavirusandthesocialimpactsondifferentethnicgroupsintheuk/2020 accessed March 2021.
10. Holden K, McGregor B, Thandi P, et al. Toward culturally centered integrative care for addressing mental health disparities among ethnic minorities. *Psychol Serv* 2014;11(4):357-68. doi: 10.1037/a0038122
11. Smith K, Bhui K, Cipriani A. COVID-19, mental health and ethnic minorities. *Evid Based Ment Health* 2020;23(3):89-90. doi: 10.1136/ebmental-2020-300174
12. Office for National Statistics. Coronavirus and the social impacts on Great Britain 2021 [Available from: https://www.ons.gov.uk/peoplepopulationandcommunity/healthandsocialcare/healthandwellbeing/bulletins/coronavirusandthesocialimpactsongreatbritain/29january2021 accessed March 2021.
13. Robinson E, Jones A, Lesser I, et al. International estimates of intended uptake and refusal of COVID-19 vaccines: A rapid systematic review and meta-analysis of large nationally representative samples. *MedRxiv* [Preprint]. 2020. (accessed March 2021).
14. Royal Society for Public Health. New poll finds ethnic minority groups less likely to want COVID vaccine 2020 [Available from: https://www.rsph.org.uk/about-us/news/new-poll-finds-bame-groups-less-likely-to-want-covid-vaccine.html accessed March 2021.
15. Lamb D, Gnanapragasam S, Greenberg N, et al. The psychosocial impact of the COVID-19 pandemic on 4,378 UK healthcare workers and ancillary staff: Initial baseline data from a cohort study collected during the first wave of the pandemic. *MedRxiv* [Preprint]. 2021.

For peer review only - http://bmjopen.bmj.com/site/about/guidelines.xhtml

16. Wise J. Covid-19: Study to assess pandemic's effects on wellbeing of NHS staff. *BMJ* 2020;371:m3942. doi: 10.1136/bmj.m3942
17. NHS England. North West London integrated care system (ICS) NHS England and NHS Improvement coronavirus. 2021 [March 2021]. Available from: https://www.england.nhs.uk/coronavirus/workforce/addressing-impact-of-covid-19-on-bame-staff-in-the-nhs/safe-working-for-all-nhs-staff/north-west-london-integrated-care-system-ics/.
18. Office for National Statistics. Ethnic group, national identity and religion 2011 [Available from: https://www.ons.gov.uk/methodology/classificationsandstandards/measuringequality/ethnicgroupnationalidentityandreligion accessed March 2021.
19. Richard B, Sivo SA, Orlowski M, et al. Qualitative Research via Focus Groups: Will Going Online Affect the Diversity of Your Findings? . *Cornell Hospitality Quarterly* 2021;62(1):32-45.
20. King N. Using templates in the thematic analysis of text. In: Symon G, Cassell C, eds. Qualitative Organisational Research: Core Methods and Current Challenges. London: Sage 2012:246-450.
21. Koelsch LE. Reconceptualising the member check interview. *International Journal of Qualitative Methods* 2013;12(1):168-79.
22. Li J-B, Yang A, Dou K, et al. Self-control moderates the association between perceived severity of coronavirus disease 2019 (COVID-19) and mental health problems among the Chinese public. *Int J Environ Res Public Health* 2020
23. Razvi S, Oliver R, Moore J, et al. Exposure of hospital healthcare workers to the novel coronavirus (SARS-CoV-2). *Clin Med* 2020;20(6):e238-e40. doi: 10.7861/clinmed.2020-0566
24. Yildirim M, Arslan G, Ozaslan A. Perceived Risk and Mental Health Problems among Healthcare Professionals during COVID-19 Pandemic: Exploring the Mediating Effects of Resilience and Coronavirus Fear. *Int J Ment Health Addict* 2020:1-11. doi: 10.1007/s11469-020-00424-8
25. Tracy DK, Tarn M, Eldridge R, et al. What should be done to support the mental health of healthcare staff treating COVID-19 patients? *Br J Psychiatry* 2020;217(4):537-39. doi: 10.1192/bjp.2020.109
26. Shah N, Ahmed IM, Nazir T. Torn Between Caution and Compassion: a Dilemma for Clinicians from Black and Minority Ethnic Groups During the COVID-19 Pandemic. *J Racial Ethn Health Disparities* 2021;8(1):21-23. doi: 10.1007/s40615-020-00938-w
27. Willan J, King AJ, Jeffery K, et al. Challenges for NHS hospitals during covid-19 epidemic. *BMJ* 2020;368:m1117. doi: 10.1136/bmj.m1117
28. Charlesworth A. Staff shortages left the NHS vulnerable to the COVID-19 storm. The Health Foundation. 2021 [May 2021]. Available from: https://www.health.org.uk/news-and-comment/blogs/staff-shortages-left-the-nhs-vulnerable-to-the-covid-19-storm.
29. Brewin CR, Fuchkan N, Huntley Z, et al. Outreach and screening following the 2005 London bombings: usage and outcomes. *Psychol Med* 2010;40(12):2049-57. doi: 10.1017/S0033291710000206
30. Brooks SK, Dunn R, Amlot R, et al. A Systematic, Thematic Review of Social and Occupational Factors Associated With Psychological Outcomes in Healthcare Employees During an Infectious Disease Outbreak. *J Occup Environ Med* 2018;60(3):248-57. doi: 10.1097/JOM.0000000000001235
31. Milligan-Saville JS, Tan L, Gayed A, et al. Workplace mental health training for managers and its effect on sick leave in employees: a cluster randomised controlled trial. *The Lancet Psychiatry* 2017;4(11):850-58. doi: 10.1016/s2215-0366(17)30372-3

For peer review only - http://bmjopen.bmj.com/site/about/guidelines.xhtml

32. Naqvi H, Wilhelm R. NHS Workforce race equality standard. 2017 data analysis report for NHS trusts 2017 [Available from: https://www.england.nhs.uk/publication/workforce-race-equality-standard-data-reporting-december-2017/ accessed March 2021.
33. Otu A, Ahinkorah BO, Ameyaw EK, et al. One country, two crises: what Covid-19 reveals about health inequalities among BAME communities in the United Kingdom and the sustainability of its health system? *Int J Equity Health* 2020;19(1):189. doi: 10.1186/s12939-020-01307-z
34. Laurencin CT, Walker JM. A Pandemic on a Pandemic: Racism and COVID-19 in Blacks. *Cell Syst* 2020;11(1):9-10. doi: 10.1016/j.cels.2020.07.002
35. Lock S, Rubin GJ, Murray V, et al. Secondary stressors and extreme events and disasters: a systematic review of primary research from 2010-2011. *PLoS Curr* 2012;4 doi: 10.1371/currents.dis.a9b76fed1b2dd5c5bfcfc13c87a2f24f
36. Tempest EL, Carter B, Beck CR, et al. Secondary stressors are associated with probable psychological morbidity after flooding: A cross-sectional analysis. *Eur J Public Health* 2017;27(6):1042-7. doi: doi:10.1093/eurpub/ckx182
37. Rana W, Mukhtar S, Mukhtar S. Mental health of medical workers in Pakistan during the pandemic COVID-19 outbreak. *Asian J Psychiatr* 2020;51:102080. doi: 10.1016/j.ajp.2020.102080
38. McCanlies EC, Gu JK, Andrew ME, et al. The effect of social support, gratitude, resilience and satisfaction with life on depressive symptoms among police officers following Hurricane Katrina. *Int J Soc Psychiatry* 2018;64(1):63-72. doi: 10.1177/0020764017746197
39. Royal College of Nursing. RCN London Research Into BAME members 2018 [Available from: https://www.rcn.org.uk/news-and-events/news/rcn-london-research-into-bame-numbers accessed March 2021.
40. Gamio L. Coronavirus worker risk. *New York Times* 15 March 2020. https://www.nytimes.com/interactive/2020/03/15/business/economy/coronavirus-worker-risk.html (accessed March 2021).
41. Tan BYQ, Chew NWS, Lee GKH, et al. Psychological Impact of the COVID-19 Pandemic on Health Care Workers in Singapore. *Ann Int Med* 2020;173(4):317-20. doi: doi:10.7326/M20-1083
42. Kapilashrami A, Bhui K. Mental health and COVID-19: is the virus racist? *Br J Psychiatry* 2020;217(2):405-07. doi: 10.1192/bjp.2020.93
43. Phiri P, Delanerolle G, Al-Sudani A, et al. COVID-19 and Black, Asian, and Minority Ethnic Communities: A Complex Relationship Without Just Cause. *JMIR Public Health Surveill* 2021;7(2):e22581. doi: 10.2196/22581
44. Pendleton J. The experiences of black and minority ethnic nurses working in the UK. *Br J Nurs* 2017;26(1):37-42.
45. Bailey S, West M. Ethnic minority deaths and Covid-19: what are we to do? The King's Fund Blog2020 [Available from: https://www.kingsfund.org.uk/blog/2020/04/ethnic-minority-deaths-covid-19 accessed March 2021.
46. Van Kerckem K. Get under their skin and in their shoes: how to address the "Race-of-interviewer effect" in qualitative research with ethnic minorities. 9th Annual conference of the European Sociological association, Abstracts, 2009.
47. White A, Strezhnev A, Lucas C, et al. Investigator Characteristics and Respondent Behavior in Online Surveys. *J Exp Polit Sci* 2018;5(1):56-67. doi: http://dx.doi.org/10.1017/XPS.2017.25

For peer review only - http://bmjopen.bmj.com/site/about/guidelines.xhtml

Figure 1: Themes and subthemes identified through thematic analysis

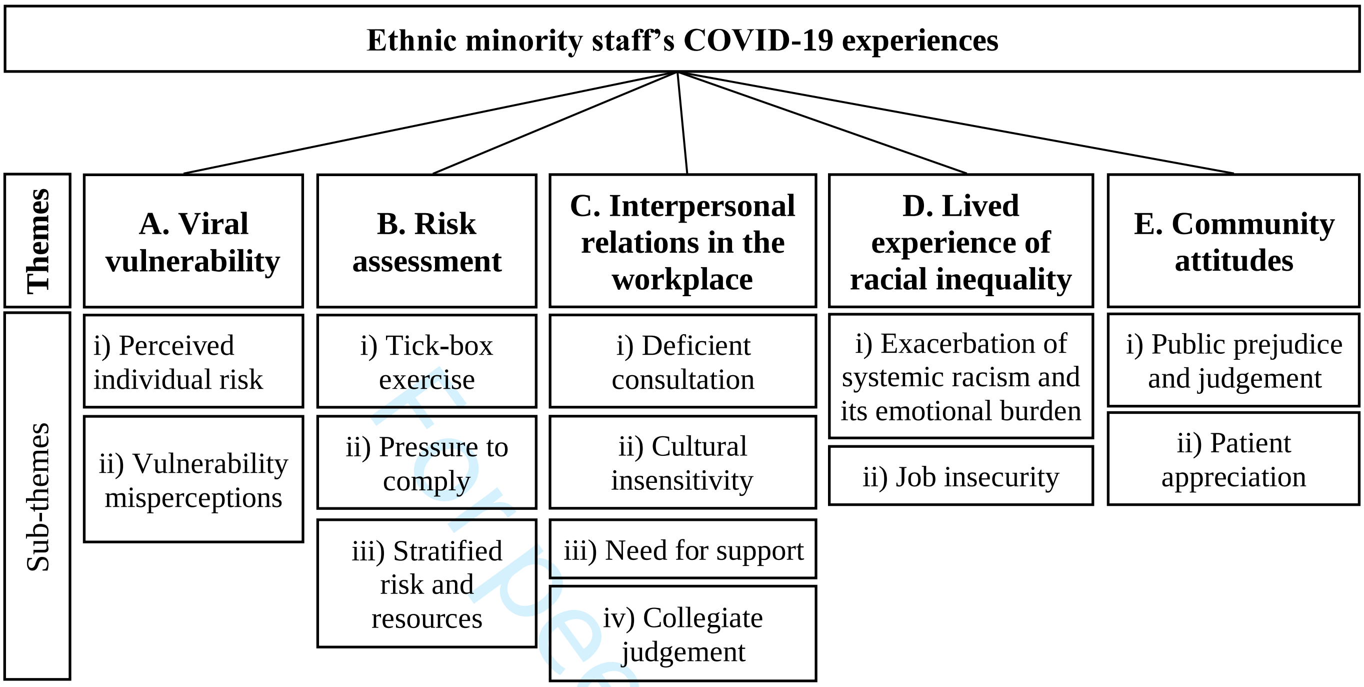

For peer review only

Focus group question guide

General

1. In general terms, how do you think being from BAME backgrounds has impacted upon your experiences during COVID?

Inside work experiences

2. Thinking of your experiences in your workplace, during COVID have you felt that you were treated differently from non-BAME staff? Is that by management and / or fellow workers? Why do you think that? Can you provide examples?

3. How do you think that your background has influenced your interactions with other staff members? What form has that taken? Why do you think that?

4. Did the way you were treated by your colleagues change during COVID compared to "normal times" (i.e., pre-COVID times)? If yes, how did it change? Can you give examples?

5. Did you notice any differences in the experiences of BAME and non-BAME staff during COVID? If yes, what form did they take?

6. Do you feel that you were given tasks/responsibilities that put you more at risk of contracting COVID than your non-BAME colleagues? If so, how did you respond in these situations? What was the subsequent response of the line manager requesting these tasks?

7. Do you feel others are responding to an aspect of your background in your dealings with them? If yes, what aspect do you think that is? (e.g., your ethnic group, physical appearance, culture, faith, etc).

8. Do you feel that your BAME background has affected the way you are treated by patients during COVID? If yes, please explain.

9. How well did you feel supported by your colleagues generally during COVID? If supported, how? If not supported, what support was absent and why do you think that was the case?

Outside work experiences

10. How has your home life changed during COVID? If yes, how exactly?

11. Has this affected your work life? If yes, how exactly?

12. Has there been an impact upon your children and other family members arising from their BAME background? If yes, please explain.

13. Have you experienced any changes in public life (e.g., on public transport) that you think is linked to your BAME background? If yes, please explain.

14. Has your work life affected your home life in any way? Please explain how.

For peer review only

15. If asking other BAME colleagues about their COVID experiences, what are the important questions to ask them?

16. Is there anything we didn't talk about which you think it would be useful to know?

For peer review only

MeCareNWL survey module on the experiences of healthcare staff from minority ethnic backgrounds

Q1 Do you feel your employer acknowledged BAME staff's increased vulnerability to COVID-19?

◯ Yes

◯ No

◯ Don't know

Q1a To what extent do you feel your employer has acknowledged BAME staff's increased vulnerability to COVID-19?

No acknowledgement — Fully acknowledged vulnerability

0 10 20 30 40 50 60 70 80 90 100

1. 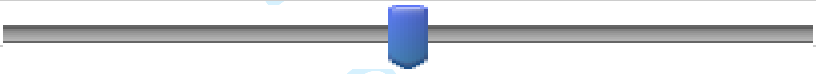

Q2 Do you feel your employer has accommodated BAME staff's increased risk from COVID-19?

◯ Yes

◯ No

◯ Don't know

For peer review only

Q2b What do you feel your employer **could have done** to accommodate BAME staff's increased risk from COVID-19?

◯ 1. ____

◯ 2. ____

◯ 3. ____

◯ 4. ____

◯ 5. ____

◯ 6. ____

◯ 7. ____

Q3 Did your employer take actions that made you feel safer in terms of your vulnerability to the virus?

◯ Yes

◯ No

◯ Not sure

Q3a What actions did your employer take that made you feel safer in terms of your vulnerability to the virus? (please select all that apply)

☐ Allowed me to work from home

☐ Provided me with adequate PPE

☐ Moved me to work in lower-risk wards / away from the front line

☐ Other (please state)

____

For peer review only

Q4 Do you feel able to speak to your manager about concerns regarding your heightened vulnerability to COVID-19 due to your ethnicity?

- ◯ Yes
- ◯ No
- ◯ Don't know

Q4a To what extent do you feel able to speak to your manager about concerns regarding your vulnerability to COVID-19?

Unable to voice my concerns — Comfortable and able to speak freely

0 10 20 30 40 50 60 70 80 90 100

1.

Q5 Did you report any concerns related to your health vulnerability associated with your ethnicity to your managers?

- ◯ Yes
- ◯ No
- ◯ Don't know

Q5a To what extent did you feel that your manager understood your concerns related to your health vulnerability associated with your ethnicity?

No understanding at all — Full understanding

0 10 20 30 40 50 60 70 80 90 100

1.

Q6 If you worked from home during COVID-19, do you feel that your BAME status affected how much your manager trusted you while you were working from home?

- ◯ Yes
- ◯ No
- ◯ Don't know
- ◯ Didn't work from home

Q6a To what extent do you feel that your manager's trust of you while working from home is due to your ethnicity?

Less trusting Because I'm BAME — More trusting because I'm BAME

0 10 20 30 40 50 60 70 80 90 100

1.

Q7 Do you feel that you have had to take risks greater than you are comfortable with?

- ◯ Yes
- ◯ No
- ◯ Don't know

Q7a How frequently did you feel you had to take risks greater than you were comfortable with in your workplace?

Never — Very frequently

0 10 20 30 40 50 60 70 80 90 100

1.

For peer review only

Q8 Did BAME staff have to work in high-risk areas in your Trust / local authority / department / practice?

○ Yes

○ No

○ Don't know

Q8a How common do you feel that the experience of BAME staff working in high-risk areas was in your Trust / local authority / department / practice?

Very rare — Very common

0 10 20 30 40 50 60 70 80 90 100

1.

Q9 Do you feel that your ethnic background played a role in your ability to obtain PPE?

○ Yes

○ No

○ Don't know

Q9a To what extent do you feel that your ethnic background has played a role in your ability to obtain PPE?

Made it harder to obtain PPE — Made it easier to obtain PPE

0 10 20 30 40 50 60 70 80 90 100

1.

For peer review only

Q10 Within your workplace, which of the following provided you with a source of support when needed? (please select all that apply)

- ☐ Manager
- ☐ BAME colleagues
- ☐ Non-BAME colleagues
- ☐ Trust staff support service
- ☐ No source of support
- ☐ Other (please specify) ________________________________

Q11 Did you feel that patients' level of appreciation for you and other BAME staff was different from their level of appreciation for non-BAME staff?

- ○ Yes
- ○ No
- ○ Don't know

Q11a How do you feel that patient's level of appreciation for staff differed between BAME staff and non-BAME staff?

	Less appreciation for BAME staff than non-BAME staff	More appreciation for BAME staff than non-BAME staff

0 10 20 30 40 50 60 70 80 90 100

1.

For peer review only

Q12 Did you **expect** to experience increased prejudice associated with your ethnic background as a result of COVID-19 **in your workplace**?

◯ Yes

◯ No

◯ Don't know

Q13 Did you **actually** experience increased prejudice associated with your ethnic background as a result of COVID-19 **in your workplace**?

◯ Yes

◯ No

◯ Don't know

Q14 Did you **expect** to experience increased prejudice associated with your ethnic background as a result of COVID-19 **in the community**?

◯ Yes

◯ No

◯ Don't know

Q15 Did you **actually** experience increased prejudice associated with your ethnic background as a result of COVID-19 **in the community**?

◯ Yes

◯ No

◯ Don't know

Q16 What practical suggestions do you have that would help you feel **heard** by management?

____

For peer review only

Q17 What practical suggestions do you have that would help you feel **safer** in your workplace?

____

For peer review only

COREQ (COnsolidated criteria for REporting Qualitative research) Checklist

A checklist of items that should be included in reports of qualitative research. You must report the page number in your manuscript where you consider each of the items listed in this checklist. If you have not included this information, either revise your manuscript accordingly before submitting or note N/A.

Topic	Item No.	Guide Questions/Description	Reported on Page No.
**Domain 1: Research team and reflexivity**			
Personal characteristics			
Interviewer/facilitator	1	Which author/s conducted the interview or focus group?	
Credentials	2	What were the researcher's credentials? E.g. PhD, MD	
Occupation	3	What was their occupation at the time of the study?	
Gender	4	Was the researcher male or female?	
Experience and training	5	What experience or training did the researcher have?	
Relationship with participants			
Relationship established	6	Was a relationship established prior to study commencement?	
Participant knowledge of the interviewer	7	What did the participants know about the researcher? e.g. personal goals, reasons for doing the research	
Interviewer characteristics	8	What characteristics were reported about the inter viewer/facilitator? e.g. Bias, assumptions, reasons and interests in the research topic	
**Domain 2: Study design**			
Theoretical framework			
Methodological orientation and Theory	9	What methodological orientation was stated to underpin the study? e.g. grounded theory, discourse analysis, ethnography, phenomenology, content analysis	
Participant selection			
Sampling	10	How were participants selected? e.g. purposive, convenience, consecutive, snowball	
Method of approach	11	How were participants approached? e.g. face-to-face, telephone, mail, email	
Sample size	12	How many participants were in the study?	
Non-participation	13	How many people refused to participate or dropped out? Reasons?	
Setting			
Setting of data collection	14	Where was the data collected? e.g. home, clinic, workplace	
Presence of non-participants	15	Was anyone else present besides the participants and researchers?	
Description of sample	16	What are the important characteristics of the sample? e.g. demographic data, date	
Data collection			
Interview guide	17	Were questions, prompts, guides provided by the authors? Was it pilot tested?	
Repeat interviews	18	Were repeat inter views carried out? If yes, how many?	
Audio/visual recording	19	Did the research use audio or visual recording to collect the data?	
Field notes	20	Were field notes made during and/or after the inter view or focus group?	
Duration	21	What was the duration of the inter views or focus group?	
Data saturation	22	Was data saturation discussed?	
Transcripts returned	23	Were transcripts returned to participants for comment and/or	

For peer review only

Topic	Item No.	Guide Questions/Description	Reported on Page No.
		correction?	
**Domain 3: analysis and findings**			
Data analysis			
Number of data coders	24	How many data coders coded the data?	
Description of the coding tree	25	Did authors provide a description of the coding tree?	
Derivation of themes	26	Were themes identified in advance or derived from the data?	
Software	27	What software, if applicable, was used to manage the data?	
Participant checking	28	Did participants provide feedback on the findings?	
Reporting			
Quotations presented	29	Were participant quotations presented to illustrate the themes/findings? Was each quotation identified? e.g. participant number	
Data and findings consistent	30	Was there consistency between the data presented and the findings?	
Clarity of major themes	31	Were major themes clearly presented in the findings?	
Clarity of minor themes	32	Is there a description of diverse cases or discussion of minor themes?	

Developed from: Tong A, Sainsbury P, Craig J. Consolidated criteria for reporting qualitative research (COREQ): a 32-item checklist for interviews and focus groups. *International Journal for Quality in Health Care*. 2007. Volume 19, Number 6: pp. 349 – 357

Once you have completed this checklist, please save a copy and upload it as part of your submission. DO NOT include this checklist as part of the main manuscript document. It must be uploaded as a separate file.

For peer review only

“We weren't checked in on, nobody spoke to us”: An exploratory qualitative analysis of two focus groups on the concerns of ethnic minority NHS staff during COVID-19

Journal:	*BMJ Open*
Manuscript ID	bmjopen-2021-053396.R2
Article Type:	Original research
Date Submitted by the Author:	25-Oct-2021

Complete List of Authors:	Jesuthasan, Jehanita; Imperial College London Faculty of Medicine, Department of Brain Sciences<br>Powell, Richard A; Imperial College London School of Public Health, Department of Primary Care and Public Health; NIHR Collaboration for Leadership in Applied Health Research and Care for Northwest London<br>Burmester, Victoria; Imperial College London Faculty of Medicine, Department of Brain Sciences<br>Nicholls, Dasha; Imperial College London Faculty of Medicine, Department of Brain Sciences; NIHR Collaboration for Leadership in Applied Health Research and Care for Northwest London

**Primary Subject Heading**:	Mental health
Secondary Subject Heading:	Health services research, Qualitative research
Keywords:	COVID-19, MENTAL HEALTH, Health & safety < HEALTH SERVICES ADMINISTRATION & MANAGEMENT, OCCUPATIONAL & INDUSTRIAL MEDICINE

SCHOLARONE™
Manuscripts

I, the Submitting Author has the right to grant and does grant on behalf of all authors of the Work (as defined in the below author licence), an exclusive licence and/or a non-exclusive licence for contributions from authors who are: i) UK Crown employees; ii) where BMJ has agreed a CC-BY licence shall apply, and/or iii) in accordance with the terms applicable for US Federal Government officers or employees acting as part of their official duties; on a worldwide, perpetual, irrevocable, royalty-free basis to BMJ Publishing Group Ltd ("BMJ") its licensees and where the relevant Journal is co-owned by BMJ to the co-owners of the Journal, to publish the Work in this journal and any other BMJ products and to exploit all rights, as set out in our licence.

The Submitting Author accepts and understands that any supply made under these terms is made by BMJ to the Submitting Author unless you are acting as an employee on behalf of your employer or a postgraduate student of an affiliated institution which is paying any applicable article publishing charge ("APC") for Open Access articles. Where the Submitting Author wishes to make the Work available on an Open Access basis (and intends to pay the relevant APC), the terms of reuse of such Open Access shall be governed by a Creative Commons licence – details of these licences and which Creative Commons licence will apply to this Work are set out in our licence referred to above.

Other than as permitted in any relevant BMJ Author's Self Archiving Policies, I confirm this Work has not been accepted for publication elsewhere, is not being considered for publication elsewhere and does not duplicate material already published. I confirm all authors consent to publication of this Work and authorise the granting of this licence.

For peer review only

"We weren't checked in on, nobody spoke to us": An exploratory qualitative analysis of two focus groups on the concerns of ethnic minority NHS staff during COVID-19

Jehanita Jesuthasan[1]
Richard A Powell[2, 3]
Victoria ML Burmester[1]
Dasha Nicholls[1, 3]

[1] Imperial College London Faculty of Medicine, Department of Brain Sciences, London, UK
[2] Imperial College London School of Public Health, Department of Primary Care and Public Health, London, UK
[3] National Institute for Health Research Collaboration for Leadership in Applied Health Research and Care for Northwest London, London, UK

Correspondence to:
Dr Dasha Nicholls
Mailing address: Department of Brain Sciences, Faculty of Medicine, Imperial College London, Floor 2, Commonwealth Building The Hammersmith Hospital, Du Cane Road, London W12 0NN
Email: d.nicholls@imperial.ac.uk
Tel: +44 (0)20 3313 8436

Word count: 4279

For peer review only - http://bmjopen.bmj.com/site/about/guidelines.xhtml

Statement of ethics approval: The MeCare study received ethical approval from the Health Research Authority (IRAS Project ID 290383), which included permission to co-create a module addressing questions specifically for ethnic minority populations with input from Patient and Public Involvement (PPI) colleagues, project partners and other experts by experience. Participants gave their informed consent before taking part.

Funding: This report is independent research funded by the National Institute for Health Research North West London Applied Research Collaboration (ARC) and by the Imperial College COVID-19 Research Fund grant number P88408. The views expressed in this publication are those of the authors and not necessarily those of the National Institute for Health Research or the Department of Health and Social Care.

Competing interests: None declared.

Data sharing statement: No additional data are available.

Author contributions: JJ, RAP, VB, and DN conceptualised the study. RAP conducted the focus groups. JJ, RAP, and VB analysed the data. JJ wrote the draft with support from RAP, VB, and DN. RAP, VB, and DN managed the overall design of the study.

Non-author contributors: John Norton and Sandra Jayacodi, partners of the National Institute for Health Research ARC Northwest London PPI Initiative, verified data interpretation and reviewed the final manuscript.

Acknowledgements: We would like to thank the participants for taking part in this research and sharing their experiences.

Figures

Figure 1: Thematic Experiences of Ethnic Minority Healthcare Staff During COVID-19

For peer review only

Supplementary Materials

1. Interview guide for the focus groups

2. Survey module created based on the focus groups

For peer review only

For peer review only - http://bmjopen.bmj.com/site/about/guidelines.xhtml

ABSTRACT

Objective

To gain exploratory insights into the multi-faceted, lived-experience impact of COVID-19 on a small sample of ethnic minority healthcare staff to co-create a module of questions for follow-up online surveys on the wellbeing of healthcare staff during the pandemic.

Design

A cross-sectional design using two online focus groups among ethnic minority healthcare workers who worked in care or supportive roles in a hospital, community health or GP surgery setting for at least 12 months.

Participants

Thirteen healthcare workers (11 female) aged 26 to 62 years from diverse ethnic minority backgrounds, eleven working in clinical roles.

Results

Five primary thematic domains emerged: 1) *viral vulnerability*, centring around perceived individual risk and vulnerability perceptions; 2) *risk assessment*, comprising of pressures to comply, perception of a tick-box exercise, and issues with risk and resource stratification; 3) *interpersonal relations in the workplace*, highlighting deficient consultation of ethnic minority staff, cultural insensitivity, need for support, and collegiate judgement; 4) *lived experience of racial inequality*, consisting of job insecurity and the exacerbation of systemic racism and its emotional burden; 5) *community attitudes*, including public prejudice and judgement, and patient appreciation.

For peer review only - http://bmjopen.bmj.com/site/about/guidelines.xhtml

For peer review only

Conclusions

Our novel study has shown ethnic minority NHS staff have experienced COVID-19 in a complex, multi-dimensional manner. Future research with a larger sample should further examine the complexity of these experiences and should enumerate the extent to which these varied thematic experiences are shared among ethnic minority NHS workers so that more empathetic and supportive management and related occupational practices can be instituted.

For peer review only

ARTICLE SUMMARY

Strengths and limitations of this study

- This is one of the first studies to examine qualitatively the experiences of ethnic minority healthcare staff during the COVID-19 pandemic.
- Due to the self-selected nature of the sample, the experiences of participants may not be representative of the ethnic minority healthcare workforce as a whole.
- Female healthcare workers were disproportionately represented in our sample.
- Given its exploratory nature, the sample size was potentially insufficient to achieve data saturation and to be representative of the diversity of experiences of ethnic minority staff in the NHS.

KEYWORDS: COVID-19; Mental Health; Occupational Health; Health and Safety

For peer review only - http://bmjopen.bmj.com/site/about/guidelines.xhtml

INTRODUCTION

COVID-19 has adversely impacted the occupational roles and physical and mental wellbeing of healthcare staff[1]. Many have experienced disrupted support structures, redeployment to areas outside their professional training[2]—sometimes engaging in tasks that transgress individual moral consciences and values[3]—limited resources and vital medical equipment[4], and unprecedented patient service demand amid rapidly changing care guidelines[5]. Staff are not only vulnerable to contracting the virus but at risk of work stress, moral injury, and mental ill-health, including clinical depression, post-traumatic stress disorder, substance misuse, and suicide[6].

Ethnic minority care staff have been disproportionately affected by the pandemic's clinical impact[7].The impact is multi-faceted: economic, social, attitudinal and cultural, as well as occupational, physical and psychological[8 9]. Understanding these broader impacts can facilitate a more holistic and empathetic understanding of the lived experiences of COVID-19 among health care staff from ethnic minorities, generating greater awareness of health-seeking behaviour in and outside the workplace, and perceived impediments to seeking and receiving support[10 11]. Increased understanding can also inform public health campaigns addressing, for example, disinclination to accept vaccines[12-14].

The MeCare study in Northwest (NW) London, collaborating with partners in the larger *NHS Check* study[15 16], is longitudinally examining the mental health and wellbeing of National Health Service (NHS) staff (including general practitioners) in NW London—where 49% of NHS staff are from ethnic minority backgrounds[17]—and exploring how to support staff during pandemics. This paper reports on the co-creation of a module of questions targeted towards staff from ethnic minority backgrounds as part of follow-up online surveys.

For peer review only - http://bmjopen.bmj.com/site/about/guidelines.xhtml

METHOD

Participants

We explored the experiences of ethnic minority healthcare workers selected through purposive sampling. Participants were recruited through Patient and Public Involvement (PPI) networks and promotional materials circulated among NHS trusts. Participants were adults from an ONS[18] ethnic minority background who worked in care or supportive roles in a hospital, community health or GP surgery setting for at least 12 months.

Before beginning, the purpose of the focus group, the purpose of recording the sessions, the anonymisation of responses for analysis and publication were reiterated. Participants were made aware that the sessions were to be recorded to facilitate subsequent analysis and informed that by attending they were agreeing to the recording of their contribution. The recordings were only to be available to the research team and were to be destroyed after they had been transcribed. Participants were also reminded that they could withdraw their participation at any time from the sessions with no consequences but that their responses up to that withdrawal point would be retained. No participants withdrew from the online meeting or afterwards; however, two participants decided not to participate on the day of the sessions: one because of IT connectivity challenges, the other because of a work emergency. Participants were offered the opportunity to ask any questions before providing their informed consent orally before taking part.

Data collection

Two 2-hour focus groups of 6 and 7 participants were held online—shown to generate idea diversity comparable to in-person equivalents[19]—using Microsoft Teams (MT) and facilitated by RAP, with participants offered the choice of having their session cameras live or not. VB was present at the beginning of both sessions to introduce the research and JJ was

For peer review only - http://bmjopen.bmj.com/site/about/guidelines.xhtml

present as a note-taker throughout both sessions. Field notes were taken by JJ on key points and the conversations were recorded using MT's software, from which manuscripts were produced. Both groups followed the study interview guide (Supplementary File 1), which was reactively adapted, in terms of questioning order and prompts. The interview guide was developed based on existing research and news reports on the experiences of ethnic minority healthcare staff in the UK during the pandemic, and upon guidance from an expert team at King's College London and independently reviewed and refined by RP, VB and JJ. In conducting the group discussions, RAP ensured each participant was able to understand and respond to each question, if they so wished. Participants also had the opportunity to respond to each other's comments and responses.

Data analysis

Discussions were transcribed verbatim and managed using MTs' automatic caption generation software and manually checked for errors. RAP, VB and JJ undertook independent content analysis using the template analysis approach[20], under a constructivist qualitative research paradigm, combining deductive and inductive analyses. The study team subsequently met to reach consensus on emergent themes and sub-themes. To ensure the veracity of data interpretation, the findings and draft survey questions were validated by member checking with a sample of group participants[21].

Patient and public involvement

Participants were recruited through PPI representatives and derived separately from the MeCare study's target population, they were not direct study participants. JN and SJ are public partners of the Applied Research Collaboration in NW London and provided feedback on data interpretation.

For peer review only - http://bmjopen.bmj.com/site/about/guidelines.xhtml

RESULTS

The final sample included 13 healthcare workers (11 female) with a mean age of 42.7 years (SD = 10.3 years). Ethnically the sample was comprised of: Eastern European (n = 1), Indian (n = 2), Mixed White and Black Caribbean (n = 1), Caribbean (n = 1), Mixed White and Asian (n = 1), African (n = 2), Arab (n = 1), Bangladeshi (n = 1), Asian Other (n = 2), and Other, in this instance Mixed North African and Eastern European, (n = 1). Eleven worked in clinical roles in primary and secondary care settings (one GP, three consultants, one nurse, one clinical lead at an urgent care team, two hospital doctors, one pharmacist, one patient housing officer, and one psychological wellbeing practitioner); two worked in administrative roles (one hospital administrator and one assistant improvement manager).

The five primary thematic domains that emerged were: viral vulnerability, risk assessment, interpersonal relationships in the workplace, lived experience of racial inequality, and community attitudes (Figure 1).

Figure 1: Themes and sub-themes identified through thematic analysis.

1) Viral vulnerability

a) Perceived individual risk

Participants were sensitive to their increased risk of infection compared to non-ethnic minority colleagues in the same roles. They discussed the impact of this heightened risk on their emotional wellbeing, reporting personal anxiety and emotional distress.

For peer review only - http://bmjopen.bmj.com/site/about/guidelines.xhtml

> P12: "I think most upsetting was the initial kind of response and risk stratifications and the deaths of people I knew or knew by indirect means."

Participants also expressed frustration that individual vulnerability was insufficiently acknowledged within their teams and organisations, a shortcoming one participant suggested was associated with the fact that it was ethnic minorities rather than the non-minority population who were affected.

> P2: "If the prevalence and incidence was affecting the White majority population more than the Black population, maybe something could be done differently."

b) Vulnerability misperceptions

Several participants expressed frustration about fundamental misunderstandings regarding the reasons for increased infection risk among ethnic minorities: in public discourse and government reports, differences in viral vulnerability were frequently attributed to individual factors (personal hygiene practices, individual obesity). Participants felt insufficient attention was paid to structural determinants, from exposure differentials to unconscious racism.

> P3: "That research was basically saying that [ethnic minorities] are at more risk because they are obese, they have got Type 2 diabetes, they're living in

For peer review only

overcrowded situations and all that. […] They did not consider that this could be just because we are exposed more.”

P13: “It's just being aware that it's not entirely biological, the risk that they face. Some of the reasons why we saw people from BAME [Black, Asian and minority ethnic] background suffer a lot more was because of their social circumstances as well.”

2) Risk assessment

a) Tick-box exercise

Many participants felt risk assessment exercises had not been treated seriously by their managers and organisations. Although some felt their employer accommodated their identified increased risk, many reported not seeing tangible protective actions for vulnerable ethnic minority staff. By not emphasising the importance of completing risk assessments, and taking minimal action following them, management made staff feel the assessment was a tick-box exercise rather than a considered strategy to reduce staff risk.

P3: “I wasn’t able to understand what the point of that risk assessment was and if somebody was found to be high risk population, whether something different was done for that person.”

For peer review only - http://bmjopen.bmj.com/site/about/guidelines.xhtml

b) Pressure to comply

Despite awareness of ethnic minority communities' COVID-19 vulnerability, participants reported feeling under pressure to continue frontline working. This arose both from managers and an innate sense of duty, with a sense of guilt associated with not seeing in-person patients among higher-risk staff members who were shielding and had moved to remote working.

> P11: "I did feel quite guilty about not being at work seeing patients. […] Every Thursday for a while [my neighbours] were all standing outside clapping and I didn't go outside. I stayed inside and hid because I felt like a complete fraud."

Other participants who were still working in person frequently felt they had little choice but to continue working in high-risk positions.

> P5: "You kind of feel you're obligated to follow through your duties, but there still is that major risk of putting yourself or your health at risk."

In terms of extrinsic pressure, the demands on NHS frontline staff limited the alternatives to positioning ethnic minority staff in frontline care provision. Consequently, many reported no choice but to maintain their position on high-risk wards—even when Personal Protective Equipment (PPE) was in short supply—and being pressured to return to work shortly after COVID-19 diagnoses.

For peer review only - http://bmjopen.bmj.com/site/about/guidelines.xhtml

For peer review only

P5: "If you pull out all the people on the frontline, then who's going to actually do the work? Who's gonna fill in those gaps?"

P12: "They were desperate to get people on the ground and there was this whole thing about 'it's a respiratory virus and it only lasts 2 weeks max'. So, many members of staff were harassed, told 'you have to go back, your two weeks are up'."

P4: "Your personal risk doesn't matter, the managers are managers, they're there to get the job done, to apply the pressure coming from above. So, if you're a nurse in a unit that is short-staffed and you turn up to work and they don't have [the necessary PPE], you either get on with your job knowing that you're at high risk, or you become a problem."

c) Stratified risk and resources

Several participants identified flaws in the risk and resultant resources stratification, feeling the organisation's workplace hierarchy was imposed on the risk assessment. They contended the distribution of PPE was based on professional hierarchy rather than individual vulnerability.

P4: "The distribution of PPE seems to have followed a hierarchical structure. So, medics are walking around with 3M rubber fit-sealed

For peer review only - http://bmjopen.bmj.com/site/about/guidelines.xhtml

masks, although they don't spend that much time in COVID areas, while some of the Filipino nurses are struggling to find a 3M mask that fits them. And then when it comes to risk assessing the domestic staff, they're an afterthought."

3) Interpersonal relations in the workplace

a) Deficient consultation

The perception of blame for increased vulnerability to COVID-19 being ascribed to ethnic minority communities and a disregard for the role of structural factors fuelled belief in a lack of understanding of ethnic minority experiences. Moreover, the view that attempts to remedy this deficiency were lacking reinforced the sense that larger racial issues were disregarded and thereby unsolved. Participants reported ethnic minorities were excluded from conversations concerning them—such as the construction of risk assessments—and felt the impacts of their vulnerability on their wellbeing were disregarded within organisations.

P1: "We weren't checked in on, nobody spoke to us to see if we were okay, how we felt about it. It just wasn't really made a big deal of. It wasn't something that mattered."

P2: "The issue of not being heard, as somebody from the BAME community. 'Cause I felt like, up to now, nobody's really asked me 'What do you want? What can we do to make you feel protected?' So, I'm aware that the risk assessment was imposed by other people from

For peer review only - http://bmjopen.bmj.com/site/about/guidelines.xhtml

other organisations. […] It was again that issue of them discussing about us without us."

b) Cultural insensitivity

The disconnect between ethnic and non-ethnic minority staff was associated with difficulties sharing concerns with non-minority supervisors. Several participants cited cultural differences and the associated lack of understanding of ethnic minority staff's experiences, needs, and problems as the principal barrier to discussing concerns with non-ethnic minority managers. Indeed, it was suggested that a common background with one's supervisor would facilitate communication and increase feelings of being supported.

> P4: "I was staying in the trust hotel and went out for a walk […] I was subsequently stopped and searched by police, assaulted. Trying to explain that to my manager resulted in the eye-rolling from them, and them not really understanding the consequences of that."

> P3: "It is always much easier to explain to people who are from your own cultural background or somebody who has experienced the same kind of thing rather than actually going to your manager who has got no idea what you're talking about."

For peer review only - http://bmjopen.bmj.com/site/about/guidelines.xhtml

For example, the difficulty obtaining hair caps, needed by ethnic minority staff who do not wash their hair daily, highlighted the impact of under-representation of such staff in senior positions and ethnic minority staff's ability to perform their jobs.

c) Need for support

Several participants stated they did not feel adequately supported by their employer and their manager during the pandemic. Some felt particularly unsupported when their supervisor was from a non-ethnic minority background, making it difficult to know their concerns related to ethnicity were heard. For participants who felt they had organisational support, it was seen as improving their wellbeing and may have mitigated the effects of racial injustices they were increasingly aware of and subjected to during the pandemic.

> P3: "Will [ethnic minority staff] feel more comfortable and more supported if they have somebody appointed that they can approach if they have any issues? […] Will they feel more comfortable if that person is from the same cultural background?"

> P11: "I think people were quite protective of me [because I'm ethnic minority] and I personally didn't find that paternalistic or patronising. I felt cared for."

d) Collegiate judgement

Several participants were wary of special treatment being accorded to them, fearing it might negatively impact how they are viewed by non-ethnic minority colleagues. It was

For peer review only - http://bmjopen.bmj.com/site/about/guidelines.xhtml

suggested some ethnic minority staff may have been reluctant to be risk-stratified and made to work from home for fear of being negatively judged by team members.

> P11: "There were a significant minority of people who, although my recommendation was that they should work from home, they chose to not follow guidance and they wanted to see patients face to face. […] I suspect that might have been because they were worried about their position in the team and about bullying."

Participants also noted that, in some cases, the special attention given to ethnic minority individuals was experienced as discrimination and a cause for bullying.

> P11: "There were a lot of people who were BAME who did feel that there was a spotlight being shone upon them. […] A lot of them didn't feel that that was positive. They felt that it could be seen as quite discriminatory, as them being given special privileges."

Moreover, several participants were defensive of the reasons for doing their jobs, emphasising their interest in, and passion for, their work and denying ulterior motives, such as hopes to receive special treatment.

> P9: "It's work that the BAME group tends to have interest in, and we do it with a love for it, rather than wanting to be specially treated. I

For peer review only - http://bmjopen.bmj.com/site/about/guidelines.xhtml

For peer review only

think sometimes when there's a lot of talk about BAME groups, it can be a little bit patronising because you feel that's a career that you've always wanted to do."

This further highlights the fear of one's job performance being judged by colleagues based on ethnicity.

4) Lived experience of racial inequality

a) Exacerbation of systemic racism and its emotional burden

There was a strong sense that existing structural workplace inequality and the emotional burden of racial injustice were exacerbated during the pandemic. Participants felt ethnic minority staff have a distinct disadvantage in career advancement, noting they had to work harder than non-minority counterparts to progress their careers. Crucially, several participants felt that increased COVID-19 vulnerability compounded career advancement challenges, and that the pandemic provided an opportunity for unconscious bias among management to manifest in career advancement opportunities given to non-ethnic minority staff.

P11: "I think that there's something similar [to being female] about being BAME compared to being White, where you know you have to work so much harder to get where you want to go to. […] Then there's COVID affecting us in a more serious way, […] it's another disadvantage."

For peer review only - http://bmjopen.bmj.com/site/about/guidelines.xhtml

> P4: “There are at least a few cases of people in the same staff group occupying two bandings. And unfortunately, BAME staff at one, slightly lower, band. And management have taken it upon themselves to use the chaos that’s going on to promote the same types of people that always get promoted.”

Additionally, participants noted the emotional burden of the pandemic is especially high among ethnic minority staff and that the Black Lives Matter movement compounded feelings of racial injustice evoked in the workplace during the pandemic and highlighted structural, societal problems.

> P1: “All of the Black Lives Matter stuff that was going on, and George Floyd and the protests, the whole attitude around them in the workplace was just exacerbating people's feelings and made it very difficult. It felt like you were being attacked from all sides.”

b) Job insecurity

Participants also reported the perception that working from home may threaten their job due to racial inequality in the workplace and that managers were less trusting of ethnic minority staff working from home than non-ethnic minority counterparts.

For peer review only - http://bmjopen.bmj.com/site/about/guidelines.xhtml

P11: “I found that the drawback [of working from home] was I wasn't getting my face seen [at work]. […] I actually almost felt that my job was threatened, I became very anxious and very insecure about my place within the team.”

P9: “[At the time I was working from home] I had a colleague I was working with who felt that that pressure [from managers] was given to the ethnic minority group because we were not trusted to be doing what we should be doing.”

5) Community attitudes

a) Public prejudice and judgement

The racial injustice experienced by ethnic minority staff in their workplace also occurred in the community, where participants reported witnessing racist slurs against East Asians and anticipating an increase in such attitudes towards themselves.

P10: “I've felt a lot more worried about having sort of racial attacks or racial comments pointed in my direction, given some of the narrative around COVID, such as the ‘Chinese Virus’.”

Additionally, the ethnicity-based judgements participants reported receiving from the public reinforced their understanding that ethnic minority staff were seen as less than non-ethnic minority colleagues in society.

For peer review only - http://bmjopen.bmj.com/site/about/guidelines.xhtml

> P5: “You feel like although you're coming to do something positive but at the same time other people might be making all sorts of judgments as to what you're doing.”

> P5: “One of my neighbours said I shouldn't be going outside, so, I said ‘No, I work for the NHS’ and automatically his response was ‘Oh! Are you a cleaner?’.”

b) Patient appreciation

Despite racial prejudices experienced in the community, participants noted patients showed an increased recognition of the value ethnic minority communities bring to the NHS and felt this increased patient appreciation was reflective of a broader attitudinal shift towards their key role.

> P6: “[Patients] were very happy to be seen, happy to be taken care of, they were politer. They really appreciated what we were doing.”

> P1: “I think there was a shift in [patients’] attitude towards NHS staff in general, which was refreshing.”

In contrast, others were sceptical of whether this recognition was indicative of a real attitudinal shift, recalling past NHS criticism. Moreover, participants highlighted a disconnect

For peer review only - http://bmjopen.bmj.com/site/about/guidelines.xhtml

between the public's appreciation of the NHS, expressed in weekly street clapping, and practical acknowledgement of the risk to which NHS staff are regularly exposed.

> P13: "When people would clap for the NHS I would think 'Oh my God they're clapping. What are they talking about? Do they even know what happens inside?'."

For peer review only

DISCUSSION

This exploratory group process examined the lived experiences of a small sample of healthcare staff from ethnic minority backgrounds during COVID-19 to develop an ethnically empathetic module for follow-up survey stages in the MeCare study (Supplementary File 2). The five primary themes that emerged from our research have highlighted key areas of concern and neglect that need to be enumerated and investigated further to appreciate the extent to which they are shared by ethnic minority staff across the NHS.

These concerns are consistent with research regarding the depression, anxiety, and stress associated with perceived COVID-19 risk and the pandemic's impact on ethnic minority communities[22-24]. The perceived pressure to work despite individual risk can significantly impact staff's work performance and mental health. For example, presenteeism—individuals presenting at work but operating sub-optimally due to health issues—can have serious consequences if it leads to poor, slow, or incorrect decision-making[25]. Moreover, Shah et al.[26] reported that during the pandemic, ethnic minority staff faced dilemmas around fulfilling their duty and continuing to deliver patient clinical care or exercising mitigating actions to avoid high-risk environments. This is especially relevant given the elevated extrinsic pressure on ethnic minority NHS frontline staff amid surges in

For peer review only - http://bmjopen.bmj.com/site/about/guidelines.xhtml

COVID-19 cases[27], combined with staffing shortages[28] and the high proportion of ethnic minority NHS staff[17].

Ethnic minority staff's mental health is likely to also be impacted by a disconnect between ethnic minority and non-minority staff. Indeed, post-trauma social support from managers influences staff's long-term mental health status[29 30], and discussion of ethnic minority staff's experiences during COVID-19 with non-minority supervisors can help foster improved attitudes towards mental health in the workplace[31]. The disconnect was manifested in fear of bullying, which is consistent with evidence that ethnic minority staff are more likely than non-minority colleagues to experience staff bullying[32 33]. Additionally, participants' sense that the pandemic exacerbated existing emotional burdens regarding systemic racism is consistent with an interplay between racism and the pandemic[34]. This may also contribute to longer-term mental ill-health—including the psychological consequences of trauma exposure[29 35 36]—among ethnic minority staff[37]. On a positive note, however, the public's acknowledgement of work undertaken by staff during the pandemic helps promote staff's resilience, thereby protecting their mental health[38].

Participants' accounts also suggest a perception that nurses and domestic staff—who are more likely to be from ethnic minority backgrounds[39] and are particularly vulnerable to infection due to their physical proximity to patients when providing care[40]—struggled to secure adequate PPE, which was more easily available for senior staff. The perception that PPE distribution was based on professional hierarchy is important. Previous research has found that, among healthcare workers, the incidence of anxiety was highest among non-medical healthcare staff, possibly because they had less first-hand information about the disease and less training on infection control measures and PPE use[41]. Additionally, adequate consultation of ethnic minority staff is especially important given they are already less likely

For peer review only - http://bmjopen.bmj.com/site/about/guidelines.xhtml

than non-minority counterparts to voice their opinions and raise concerns about inadequate PPE, increasing the likelihood of their needs being overlooked[33 42].

Racial inequalities were also reported in career advancement and treatment by managers. This is consistent with reports that ethnic minority staff have been restricted to certain NHS roles due to inequalities in career development and workplace and societal discrimination, rendering them more likely to work in critical specialties and services during the pandemic[43]. Moreover, ethnic minority staff are also more likely than non-ethnic minority staff to experience excessive scrutiny and punishment[44] and discrimination[45] from their managers.

In terms of study limitations, an important limitation was that the sample was small and self-selected. As such, the findings may not fully capture the complexity and multi-faceted nature of ethnic minority healthcare staff during the COVID-19 pandemic, and participants were likely to feel more strongly about their experience of the pandemic compared to the ethnic minority workforce as a whole. However, participants were purposively sought for their opinions to gain broad insights into this neglected area to inform further inquiry, rather than generating representative data. The MeCare module that this research informed will provide an opportunity to collect data from a broader range of ethnic minority staff and enable exploration of whether these experiences were representative and resonate with ethnic minority staff.

Second, female healthcare workers were disproportionately represented in our sample, potentially omitting specifically male experiences during the pandemic. Third, there is the possibility that the facilitator's race (non-minority ethnic) biased the data by inhibiting open discussion[46]. However, the facilitator has over 20 years' experience of health research in diverse settings including low- and middle-income countries, and JJ, who was present in both focus groups, is minority ethnic. In addition, discussions were frank and unimpeded, and

For peer review only - http://bmjopen.bmj.com/site/about/guidelines.xhtml

evidence indicates the impacts of the interviewer's ethnicity are minimal[47]. Fourth, the sample size was potentially insufficient to achieve thematic saturation as this was an exploratory exercise to highlight largely unexamined thematic areas that are currently being further researched. To minimize the possibility of the authors' invalid interpretation of the data, the study used respondent validation among a sample of group participants.

Our novel study has clearly shown that ethnic minority NHS staff have experienced COVID-19 in a complex, multi-dimensional manner. The physical and psychological impact of the virus has been overlayed by wider occupational, attitudinal (including anticipatory racism) and socio-cultural experiences. A need exists to understand the extent to which these varied experiences are shared among ethnic minority NHS workers so more empathetic and supportive management and related occupational practices can be instituted.

For peer review only

For peer review only - http://bmjopen.bmj.com/site/about/guidelines.xhtml

References

1. Gilleen J, Santaolalla A, Valdearenas L, et al. The impact of the COVID-19 pandemic on the mental health and wellbeing of UK healthcare workers. *MedRxiv* [Preprint]. 2020. (accessed March 2021).
2. Vera San Juan N, Camilleri M, Jeans JP, et al. Redeployment and training of healthcare professionals to Intensive Care during COVID-19: a systemic review. *MedRxiv* [Preprint]. 2021. (accessed March 2021).
3. Newdick C, Sheehan M, Dunn M. Tragic choices in intensive care during the COVID-19 pandemic: on fairness, consistency and community. *J Med Ethics* 2020;46(10):646-51. doi: 10.1136/medethics-2020-106487
4. Supady A, Curtis JR, Abrams D, et al. Allocating scarce intensive care resources during the COVID-19 pandemic: practical challenges to theoretical frameworks. *The Lancet Respiratory Medicine* 2021;9(4):430-34. doi: 10.1016/s2213-2600(20)30580-4
5. Vindrola-Padros C, Andrews L, Dowrick A, et al. Perceptions and experiences of healthcare workers during the COVID-19 pandemic in the UK. *BMJ Open* 2020;10(11):e040503. doi: 10.1136/bmjopen-2020-040503
6. Greenberg N. Mental health of health-care workers in the COVID-19 era. *Nat Rev Nephrol* 2020;16(8):425-26. doi: 10.1038/s41581-020-0314-5
7. Shields A, Faustini SE, Perez-Toledo M, et al. SARS-CoV-2 seroprevalence and asymptomatic viral carriage in healthcare workers: a cross-sectional study. *Thorax* 2020;75(12):1089-94. doi: 10.1136/thoraxjnl-2020-215414
8. Jaspal R, Lopes B. Discrimination and mental health outcomes in British Black and South Asian people during the COVID-19 outbreak in the UK. *Mental Health, Religion & Culture* 2021;24(1):80-96. doi: 10.1080/13674676.2020.1871328
9. Office for National Statistics. Coronavirus and the social impacts on different ethnic groups in the UK: 2020 2020 [Available from: https://www.ons.gov.uk/peoplepopulationandcommunity/culturalidentity/ethnicity/articles/coronavirusandthesocialimpactsondifferentethnicgroupsintheuk/2020 accessed March 2021.
10. Holden K, McGregor B, Thandi P, et al. Toward culturally centered integrative care for addressing mental health disparities among ethnic minorities. *Psychol Serv* 2014;11(4):357-68. doi: 10.1037/a0038122
11. Smith K, Bhui K, Cipriani A. COVID-19, mental health and ethnic minorities. *Evid Based Ment Health* 2020;23(3):89-90. doi: 10.1136/ebmental-2020-300174
12. Office for National Statistics. Coronavirus and the social impacts on Great Britain 2021 [Available from: https://www.ons.gov.uk/peoplepopulationandcommunity/healthandsocialcare/healthandwellbeing/bulletins/coronavirusandthesocialimpactsongreatbritain/29january2021 accessed March 2021.
13. Robinson E, Jones A, Lesser I, et al. International estimates of intended uptake and refusal of COVID-19 vaccines: A rapid systematic review and meta-analysis of large nationally representative samples. *MedRxiv* [Preprint]. 2020. (accessed March 2021).
14. Royal Society for Public Health. New poll finds ethnic minority groups less likely to want COVID vaccine 2020 [Available from: https://www.rsph.org.uk/about-us/news/new-poll-finds-bame-groups-less-likely-to-want-covid-vaccine.html accessed March 2021.
15. Lamb D, Gnanapragasam S, Greenberg N, et al. The psychosocial impact of the COVID-19 pandemic on 4,378 UK healthcare workers and ancillary staff: Initial baseline data from a cohort study collected during the first wave of the pandemic. *MedRxiv* [Preprint]. 2021.

For peer review only - http://bmjopen.bmj.com/site/about/guidelines.xhtml

16. Wise J. Covid-19: Study to assess pandemic's effects on wellbeing of NHS staff. *BMJ* 2020;371:m3942. doi: 10.1136/bmj.m3942
17. NHS England. North West London integrated care system (ICS) NHS England and NHS Improvement coronavirus. 2021 [March 2021]. Available from: https://www.england.nhs.uk/coronavirus/workforce/addressing-impact-of-covid-19-on-bame-staff-in-the-nhs/safe-working-for-all-nhs-staff/north-west-london-integrated-care-system-ics/.
18. Office for National Statistics. Ethnic group, national identity and religion 2011 [Available from: https://www.ons.gov.uk/methodology/classificationsandstandards/measuringequality/ethnicgroupnationalidentityandreligion accessed March 2021.
19. Richard B, Sivo SA, Orlowski M, et al. Qualitative Research via Focus Groups: Will Going Online Affect the Diversity of Your Findings? . *Cornell Hospitality Quarterly* 2021;62(1):32-45.
20. King N. Using templates in the thematic analysis of text. In: Symon G, Cassell C, eds. Qualitative Organisational Research: Core Methods and Current Challenges. London: Sage 2012:246-450.
21. Koelsch LE. Reconceptualising the member check interview. *International Journal of Qualitative Methods* 2013;12(1):168-79.
22. Li J-B, Yang A, Dou K, et al. Self-control moderates the association between perceived severity of coronavirus disease 2019 (COVID-19) and mental health problems among the Chinese public. *Int J Environ Res Public Health* 2020
23. Razvi S, Oliver R, Moore J, et al. Exposure of hospital healthcare workers to the novel coronavirus (SARS-CoV-2). *Clin Med* 2020;20(6):e238-e40. doi: 10.7861/clinmed.2020-0566
24. Yildirim M, Arslan G, Ozaslan A. Perceived Risk and Mental Health Problems among Healthcare Professionals during COVID-19 Pandemic: Exploring the Mediating Effects of Resilience and Coronavirus Fear. *Int J Ment Health Addict* 2020:1-11. doi: 10.1007/s11469-020-00424-8
25. Tracy DK, Tarn M, Eldridge R, et al. What should be done to support the mental health of healthcare staff treating COVID-19 patients? *Br J Psychiatry* 2020;217(4):537-39. doi: 10.1192/bjp.2020.109
26. Shah N, Ahmed IM, Nazir T. Torn Between Caution and Compassion: a Dilemma for Clinicians from Black and Minority Ethnic Groups During the COVID-19 Pandemic. *J Racial Ethn Health Disparities* 2021;8(1):21-23. doi: 10.1007/s40615-020-00938-w
27. Willan J, King AJ, Jeffery K, et al. Challenges for NHS hospitals during covid-19 epidemic. *BMJ* 2020;368:m1117. doi: 10.1136/bmj.m1117
28. Charlesworth A. Staff shortages left the NHS vulnerable to the COVID-19 storm. The Health Foundation. 2021 [May 2021]. Available from: https://www.health.org.uk/news-and-comment/blogs/staff-shortages-left-the-nhs-vulnerable-to-the-covid-19-storm.
29. Brewin CR, Fuchkan N, Huntley Z, et al. Outreach and screening following the 2005 London bombings: usage and outcomes. *Psychol Med* 2010;40(12):2049-57. doi: 10.1017/S0033291710000206
30. Brooks SK, Dunn R, Amlot R, et al. A Systematic, Thematic Review of Social and Occupational Factors Associated With Psychological Outcomes in Healthcare Employees During an Infectious Disease Outbreak. *J Occup Environ Med* 2018;60(3):248-57. doi: 10.1097/JOM.0000000000001235
31. Milligan-Saville JS, Tan L, Gayed A, et al. Workplace mental health training for managers and its effect on sick leave in employees: a cluster randomised controlled trial. *The Lancet Psychiatry* 2017;4(11):850-58. doi: 10.1016/s2215-0366(17)30372-3

For peer review only - http://bmjopen.bmj.com/site/about/guidelines.xhtml

32. Naqvi H, Wilhelm R. NHS Workforce race equality standard. 2017 data analysis report for NHS trusts 2017 [Available from: https://www.england.nhs.uk/publication/workforce-race-equality-standard-data-reporting-december-2017/ accessed March 2021.
33. Otu A, Ahinkorah BO, Ameyaw EK, et al. One country, two crises: what Covid-19 reveals about health inequalities among BAME communities in the United Kingdom and the sustainability of its health system? *Int J Equity Health* 2020;19(1):189. doi: 10.1186/s12939-020-01307-z
34. Laurencin CT, Walker JM. A Pandemic on a Pandemic: Racism and COVID-19 in Blacks. *Cell Syst* 2020;11(1):9-10. doi: 10.1016/j.cels.2020.07.002
35. Lock S, Rubin GJ, Murray V, et al. Secondary stressors and extreme events and disasters: a systematic review of primary research from 2010-2011. *PLoS Curr* 2012;4 doi: 10.1371/currents.dis.a9b76fed1b2dd5c5bfcfc13c87a2f24f
36. Tempest EL, Carter B, Beck CR, et al. Secondary stressors are associated with probable psychological morbidity after flooding: A cross-sectional analysis. *Eur J Public Health* 2017;27(6):1042-7. doi: doi:10.1093/eurpub/ckx182
37. Rana W, Mukhtar S, Mukhtar S. Mental health of medical workers in Pakistan during the pandemic COVID-19 outbreak. *Asian J Psychiatr* 2020;51:102080. doi: 10.1016/j.ajp.2020.102080
38. McCanlies EC, Gu JK, Andrew ME, et al. The effect of social support, gratitude, resilience and satisfaction with life on depressive symptoms among police officers following Hurricane Katrina. *Int J Soc Psychiatry* 2018;64(1):63-72. doi: 10.1177/0020764017746197
39. Royal College of Nursing. RCN London Research Into BAME members 2018 [Available from: https://www.rcn.org.uk/news-and-events/news/rcn-london-research-into-bame-numbers accessed March 2021.
40. Gamio L. Coronavirus worker risk. *New York Times* 15 March 2020. https://www.nytimes.com/interactive/2020/03/15/business/economy/coronavirus-worker-risk.html (accessed March 2021).
41. Tan BYQ, Chew NWS, Lee GKH, et al. Psychological Impact of the COVID-19 Pandemic on Health Care Workers in Singapore. *Ann Int Med* 2020;173(4):317-20. doi: doi:10.7326/M20-1083
42. Kapilashrami A, Bhui K. Mental health and COVID-19: is the virus racist? *Br J Psychiatry* 2020;217(2):405-07. doi: 10.1192/bjp.2020.93
43. Phiri P, Delanerolle G, Al-Sudani A, et al. COVID-19 and Black, Asian, and Minority Ethnic Communities: A Complex Relationship Without Just Cause. *JMIR Public Health Surveill* 2021;7(2):e22581. doi: 10.2196/22581
44. Pendleton J. The experiences of black and minority ethnic nurses working in the UK. *Br J Nurs* 2017;26(1):37-42.
45. Bailey S, West M. Ethnic minority deaths and Covid-19: what are we to do? The King's Fund Blog2020 [Available from: https://www.kingsfund.org.uk/blog/2020/04/ethnic-minority-deaths-covid-19 accessed March 2021.
46. Van Kerckem K. Get under their skin and in their shoes: how to address the "Race-of-interviewer effect" in qualitative research with ethnic minorities. 9th Annual conference of the European Sociological association, Abstracts, 2009.
47. White A, Strezhnev A, Lucas C, et al. Investigator Characteristics and Respondent Behavior in Online Surveys. *J Exp Polit Sci* 2018;5(1):56-67. doi: http://dx.doi.org/10.1017/XPS.2017.25

For peer review only - http://bmjopen.bmj.com/site/about/guidelines.xhtml

Figure 1: Themes and subthemes identified through thematic analysis

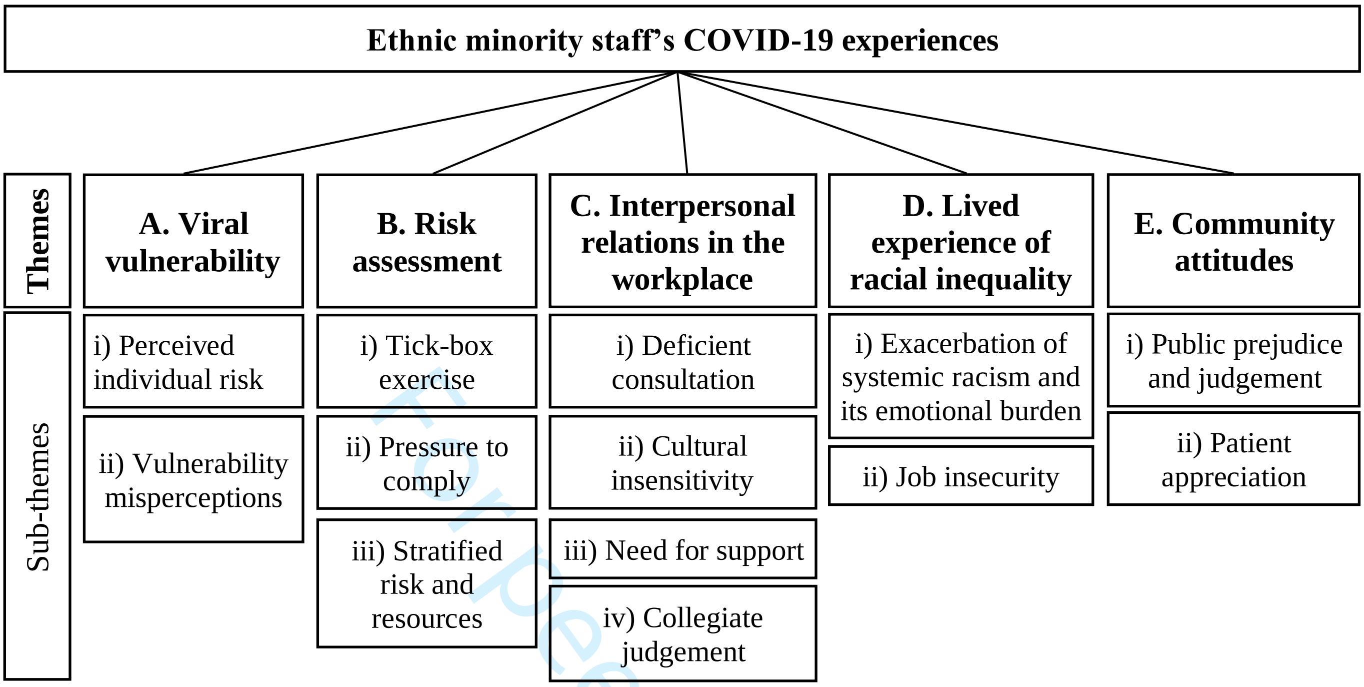

For peer review only

Focus group question guide

General

1. In general terms, how do you think being from BAME backgrounds has impacted upon your experiences during COVID?

Inside work experiences

2. Thinking of your experiences in your workplace, during COVID have you felt that you were treated differently from non-BAME staff? Is that by management and / or fellow workers? Why do you think that? Can you provide examples?

3. How do you think that your background has influenced your interactions with other staff members? What form has that taken? Why do you think that?

4. Did the way you were treated by your colleagues change during COVID compared to "normal times" (i.e., pre-COVID times)? If yes, how did it change? Can you give examples?

5. Did you notice any differences in the experiences of BAME and non-BAME staff during COVID? If yes, what form did they take?

6. Do you feel that you were given tasks/responsibilities that put you more at risk of contracting COVID than your non-BAME colleagues? If so, how did you respond in these situations? What was the subsequent response of the line manager requesting these tasks?

7. Do you feel others are responding to an aspect of your background in your dealings with them? If yes, what aspect do you think that is? (e.g., your ethnic group, physical appearance, culture, faith, etc).

8. Do you feel that your BAME background has affected the way you are treated by patients during COVID? If yes, please explain.

9. How well did you feel supported by your colleagues generally during COVID? If supported, how? If not supported, what support was absent and why do you think that was the case?

Outside work experiences

10. How has your home life changed during COVID? If yes, how exactly?

11. Has this affected your work life? If yes, how exactly?

12. Has there been an impact upon your children and other family members arising from their BAME background? If yes, please explain.

13. Have you experienced any changes in public life (e.g., on public transport) that you think is linked to your BAME background? If yes, please explain.

14. Has your work life affected your home life in any way? Please explain how.

For peer review only

15. If asking other BAME colleagues about their COVID experiences, what are the important questions to ask them?

16. Is there anything we didn't talk about which you think it would be useful to know?

For peer review only

MeCareNWL survey module on the experiences of healthcare staff from minority ethnic backgrounds

Q1 Do you feel your employer acknowledged BAME staff's increased vulnerability to COVID-19?

- ◯ Yes
- ◯ No
- ◯ Don't know

Q1a To what extent do you feel your employer has acknowledged BAME staff's increased vulnerability to COVID-19?

	No acknowledgement	Fully acknowledged vulnerability
	0 10 20 30 40 50 60 70 80 90 100	
1.		

Q2 Do you feel your employer has accommodated BAME staff's increased risk from COVID-19?

- ◯ Yes
- ◯ No
- ◯ Don't know

For peer review only

Q2b What do you feel your employer **could have done** to accommodate BAME staff's increased risk from COVID-19?

◯ 1. ____

◯ 2. ____

◯ 3. ____

◯ 4. ____

◯ 5. ____

◯ 6. ____

◯ 7. ____

Q3 Did your employer take actions that made you feel safer in terms of your vulnerability to the virus?

◯ Yes

◯ No

◯ Not sure

Q3a What actions did your employer take that made you feel safer in terms of your vulnerability to the virus? (please select all that apply)

☐ Allowed me to work from home

☐ Provided me with adequate PPE

☐ Moved me to work in lower-risk wards / away from the front line

☐ Other (please state)

____

For peer review only

Q4 Do you feel able to speak to your manager about concerns regarding your heightened vulnerability to COVID-19 due to your ethnicity?

- ◯ Yes
- ◯ No
- ◯ Don't know

Q4a To what extent do you feel able to speak to your manager about concerns regarding your vulnerability to COVID-19?

Unable to voice my concerns — Comfortable and able to speak freely

0 10 20 30 40 50 60 70 80 90 100

1.

Q5 Did you report any concerns related to your health vulnerability associated with your ethnicity to your managers?

- ◯ Yes
- ◯ No
- ◯ Don't know

Q5a To what extent did you feel that your manager understood your concerns related to your health vulnerability associated with your ethnicity?

No understanding at all — Full understanding

0 10 20 30 40 50 60 70 80 90 100

1.

For peer review only

Q6 If you worked from home during COVID-19, do you feel that your BAME status affected how much your manager trusted you while you were working from home?

- ○ Yes
- ○ No
- ○ Don't know
- ○ Didn't work from home

Q6a To what extent do you feel that your manager's trust of you while working from home is due to your ethnicity?

Less trusting Because I'm BAME — More trusting because I'm BAME

0 10 20 30 40 50 60 70 80 90 100

1.

Q7 Do you feel that you have had to take risks greater than you are comfortable with?

- ○ Yes
- ○ No
- ○ Don't know

Q7a How frequently did you feel you had to take risks greater than you were comfortable with in your workplace?

Never — Very frequently

0 10 20 30 40 50 60 70 80 90 100

1.

For peer review only

Q8 Did BAME staff have to work in high-risk areas in your Trust / local authority / department / practice?

- ◯ Yes
- ◯ No
- ◯ Don't know

Q8a How common do you feel that the experience of BAME staff working in high-risk areas was in your Trust / local authority / department / practice?

Very rare — Very common

0 10 20 30 40 50 60 70 80 90 100

1.

For peer review only

Q9 Do you feel that your ethnic background played a role in your ability to obtain PPE?

- ◯ Yes
- ◯ No
- ◯ Don't know

Q9a To what extent do you feel that your ethnic background has played a role in your ability to obtain PPE?

Made it harder to obtain PPE — Made it easier to obtain PPE

0 10 20 30 40 50 60 70 80 90 100

1.

Q10 Within your workplace, which of the following provided you with a source of support when needed? (please select all that apply)

- ☐ Manager
- ☐ BAME colleagues
- ☐ Non-BAME colleagues
- ☐ Trust staff support service
- ☐ No source of support
- ☐ Other (please specify) ____

Q11 Did you feel that patients' level of appreciation for you and other BAME staff was different from their level of appreciation for non-BAME staff?

- ○ Yes
- ○ No
- ○ Don't know

Q11a How do you feel that patient's level of appreciation for staff differed between BAME staff and non-BAME staff?

	Less appreciation for BAME staff than non-BAME staff									More appreciation for BAME staff than non-BAME staff
	0	10	20	30	40	50	60	70	80	90	100
1.											

For peer review only

Q12 Did you **expect** to experience increased prejudice associated with your ethnic background as a result of COVID-19 **in your workplace**?

- ◯ Yes
- ◯ No
- ◯ Don't know

Q13 Did you **actually** experience increased prejudice associated with your ethnic background as a result of COVID-19 **in your workplace**?

- ◯ Yes
- ◯ No
- ◯ Don't know

Q14 Did you **expect** to experience increased prejudice associated with your ethnic background as a result of COVID-19 **in the community**?

- ◯ Yes
- ◯ No
- ◯ Don't know

Q15 Did you **actually** experience increased prejudice associated with your ethnic background as a result of COVID-19 **in the community**?

- ◯ Yes
- ◯ No
- ◯ Don't know

Q16 What practical suggestions do you have that would help you feel **heard** by management?

____

For peer review only

Q17 What practical suggestions do you have that would help you feel **safer** in your workplace?

____

For peer review only

COREQ (COnsolidated criteria for REporting Qualitative research) Checklist

A checklist of items that should be included in reports of qualitative research. You must report the page number in your manuscript where you consider each of the items listed in this checklist. If you have not included this information, either revise your manuscript accordingly before submitting or note N/A.

Topic	Item No.	Guide Questions/Description	Reported on Page No.
**Domain 1: Research team and reflexivity**			
Personal characteristics			
Interviewer/facilitator	1	Which author/s conducted the interview or focus group?	
Credentials	2	What were the researcher's credentials? E.g. PhD, MD	
Occupation	3	What was their occupation at the time of the study?	
Gender	4	Was the researcher male or female?	
Experience and training	5	What experience or training did the researcher have?	
Relationship with participants			
Relationship established	6	Was a relationship established prior to study commencement?	
Participant knowledge of the interviewer	7	What did the participants know about the researcher? e.g. personal goals, reasons for doing the research	
Interviewer characteristics	8	What characteristics were reported about the inter viewer/facilitator? e.g. Bias, assumptions, reasons and interests in the research topic	
**Domain 2: Study design**			
Theoretical framework			
Methodological orientation and Theory	9	What methodological orientation was stated to underpin the study? e.g. grounded theory, discourse analysis, ethnography, phenomenology, content analysis	
Participant selection			
Sampling	10	How were participants selected? e.g. purposive, convenience, consecutive, snowball	
Method of approach	11	How were participants approached? e.g. face-to-face, telephone, mail, email	
Sample size	12	How many participants were in the study?	
Non-participation	13	How many people refused to participate or dropped out? Reasons?	
Setting			
Setting of data collection	14	Where was the data collected? e.g. home, clinic, workplace	
Presence of non-participants	15	Was anyone else present besides the participants and researchers?	
Description of sample	16	What are the important characteristics of the sample? e.g. demographic data, date	
Data collection			
Interview guide	17	Were questions, prompts, guides provided by the authors? Was it pilot tested?	
Repeat interviews	18	Were repeat inter views carried out? If yes, how many?	
Audio/visual recording	19	Did the research use audio or visual recording to collect the data?	
Field notes	20	Were field notes made during and/or after the inter view or focus group?	
Duration	21	What was the duration of the inter views or focus group?	
Data saturation	22	Was data saturation discussed?	
Transcripts returned	23	Were transcripts returned to participants for comment and/or	

For peer review only

Topic	Item No.	Guide Questions/Description	Reported on Page No.
		correction?	
**Domain 3: analysis and findings**			
Data analysis			
Number of data coders	24	How many data coders coded the data?	
Description of the coding tree	25	Did authors provide a description of the coding tree?	
Derivation of themes	26	Were themes identified in advance or derived from the data?	
Software	27	What software, if applicable, was used to manage the data?	
Participant checking	28	Did participants provide feedback on the findings?	
Reporting			
Quotations presented	29	Were participant quotations presented to illustrate the themes/findings? Was each quotation identified? e.g. participant number	
Data and findings consistent	30	Was there consistency between the data presented and the findings?	
Clarity of major themes	31	Were major themes clearly presented in the findings?	
Clarity of minor themes	32	Is there a description of diverse cases or discussion of minor themes?	

Developed from: Tong A, Sainsbury P, Craig J. Consolidated criteria for reporting qualitative research (COREQ): a 32-item checklist for interviews and focus groups. *International Journal for Quality in Health Care*. 2007. Volume 19, Number 6: pp. 349 – 357

Once you have completed this checklist, please save a copy and upload it as part of your submission. DO NOT include this checklist as part of the main manuscript document. It must be uploaded as a separate file.

For peer review only
